# Evolutionary and Predictive Functional Insights into the Aquaporin Gene Family in the Allotetraploid Plant *Nicotiana tabacum*

**DOI:** 10.3390/ijms21134743

**Published:** 2020-07-03

**Authors:** Jahed Ahmed, Sébastien Mercx, Marc Boutry, François Chaumont

**Affiliations:** Louvain Institute of Biomolecular Science and Technology, UCLouvain, Croix du Sud 4-L7.07.14, B-1348 Louvain-la-Neuve, Belgium; jahed.ahmed@uclouvain.be (J.A.); mercxsebastien@gmail.com (S.M.); marc.boutry@uclouvain.be (M.B.)

**Keywords:** aquaporins, bright yellow-2 suspension cells, *Nicotiana tabacum*, substrate specificity, phylogeny

## Abstract

Aquaporins (AQPs) are a class of integral membrane proteins that facilitate the membrane diffusion of water and other small solutes. *Nicotiana tabacum* is an important model plant, and its allotetraploid genome has recently been released, providing us with the opportunity to analyze the *AQP* gene family and its evolution. A total of 88 full-length *AQP* genes were identified in the *N. tabacum* genome, and the encoding proteins were assigned into five subfamilies: 34 plasma membrane intrinsic proteins (PIPs); 27 tonoplast intrinsic proteins (TIPs); 20 nodulin26-like intrinsic proteins (NIPs); 3 small basic intrinsic proteins (SIPs); 4 uncharacterized X intrinsic proteins (XIPs), including two splice variants. We also analyzed the genomes of two *N. tabacum* ancestors, *Nicotiana* *tomentosiformis* and *Nicotiana* *sylvestris,* and identified 49 *AQP* genes in each species. Functional prediction, based on the substrate specificity-determining positions (SDPs), revealed significant differences in substrate specificity among the AQP subfamilies. Analysis of the organ-specific *AQP* expression levels in the *N. tabacum* plant and RNA-seq data of *N. tabacum* bright yellow-2 suspension cells indicated that many AQPs are simultaneously expressed, but differentially, according to the organs or the cells. Altogether, these data constitute an important resource for future investigations of the molecular, evolutionary, and physiological functions of AQPs in *N. tabacum*.

## 1. Introduction

Aquaporins (AQPs), also known as major intrinsic proteins (MIPs), are small integral membrane proteins present in almost all living organisms [1,2]. Plants maintain a large and diverse AQP family compared to mammals. For instance, the genomes of rice (*Oryza sativa)*, Arabidopsis (*Arabidopsis thaliana)*, maize (*Zea mays*), soybean (*Glycine max*), switchgrass (*Panicum virgatum*), foxtail millet (*Setaria italica*), sorghum (*Sorghum bicolor*), *Brachypodium distachyon,* tomato (*Solanum lycopersicum*), poplar (*Populus trichocarpa*), cotton (*Gossypium hirsutum*), and potato (*Solanum tuberosum*) encode 39, 35, 36, 66, 68, 42, 38, 28, 47, 55, 71, and 41 AQP homologs, respectively [3,4,5,6,7,8,9,10,11,12], compared to only 13 *AQP* genes in mammals [13]. Based on phylogenetic analysis and subcellular localization, vascular plant AQPs are categorized into five subfamilies: (1) plasma membrane intrinsic proteins (PIPs); (2) tonoplast intrinsic proteins (TIPs); (3) nodulin-26-like intrinsic proteins (NIPs); (4) small basic intrinsic proteins (SIPs); (5) uncharacterized X intrinsic proteins (XIPs). To date, the latter subfamily has not been found in Brassicaceae and in monocots [14,15].

While many plant AQPs primarily function as water channels, they can also transport a wide range of substrates, such as ammonia (NH_3_), antimony (Sb), arsenic (As), boron (B), glycerol, hydrogen peroxide (H_2_O_2_), silicon (Si), and urea (U) [2,16,17,18,19]. Furthermore, some AQPs facilitate gas diffusion, such as carbon dioxide (CO_2_) and oxygen (O_2_) [20,21,22]. Recently it was reported that AtPIP2;1 has cations (Na^+^ and K^+^) channel activity [23]. AQPs from various plants are also involved in transmembrane water conductance in numerous physiological processes, such as cell water homeostasis, root water uptake from the soil, root and leaf hydraulic conductance, lateral root emergence, motor cell movement, rapid internode elongation, the diurnal regulation of leaf movements, and petal development and movement [1,2,24,25,26,27,28,29].

The AQP structure comprises six transmembrane (TM) α-helices (TM1-TM6), which are linked by five loops (loops A–E) and two highly conserved NPA (Asn-Pro-Ala) motifs. They form homo- or hetero-tetrameric complexes in which each subunit acts as a functional water channel [2,30]. The channel pore contains two constriction regions that contribute to the transport selectivity. The first constriction is formed at the pore center by two highly conserved NPA motifs [31]. The second constriction is the aromatic/arginine (ar/R) filter, formed at the extracellular aperture of the pore by four residues from TM2, TM5, and loop E (LE1 and LE2), respectively [32,33]. Additionally, five amino acid residues known as Froger’s positions (FPs) designated P1–P5, are also associated with substrate selectivity [34,35]. More recently, some substrate specificity determining positions (SDPs) have been proposed for B, H_2_O_2_, CO_2_, NH_3_, As, Sb, and Si [9,17].

*Nicotiana tabacum* (tobacco)*,* a perennial herbaceous plant of the Solanaceae family, is an allotetraploid (2n = 4x = 48), which evolved by the natural hybridization of the ancestors of *Nicotiana sylvestris* (2n = 24, maternal donor) and *Nicotiana tomentosiformis* (2n = 24, paternal donor) about 200,000 years ago [36,37]. *N. tabacum* is intensively studied as a versatile model organism for understanding genetics, functional genomics, cellular and molecular biology, biochemistry and physiology [38]. In this study, we identified *AQP* genes in the genomes of *N. tabacum* as well as its two ancestors, *N. tomentosiformis* and *N. sylvestris*, and analyzed the transcriptome data of *N. tabacum* plant and Bright Yellow-2 (BY-2) suspension cells [39]. We investigated the phylogenetic relationships, as well as the structural properties and subcellular localization of AQPs in *N. tabacum*. Comparing the primary selectivity motifs, we further predicted their probable substrate transport activities. Altogether, this study provides new insights into the expression patterns in different organs and suspension cells, as well as the transmembrane transport selectivity of AQPs in *N. tabacum*.

## 2. Results

### 2.1. Genome-Wide Identification and Characterization of NtAQP Genes

The whole genome shotgun sequence of *N. tabacum* and its two ancestors, *N. tomentosiformis* and *N. sylvestris*, were searched for *AQP* genes, using pBLAST and AQP sequences from *S. tuberosum* and *S. lycopersicum* as queries. NtAQP protein sequences were analyzed and compared with SlAQP and StAQP for domain identification and functional annotation. Of 101 initial unique hits for *NtAQPs*, 13 were considered *AQP* pseudogenes and discarded after a manual inspection of their nucleotide and amino acid sequences and their TM domains. We finally obtained 88 genes encoding 90 full-length AQP proteins, and *NtXIP1;1* and *NtXIP1;2* genes encoding two splice variants (α and β), as shown in Table 1.

**Figure 1:**
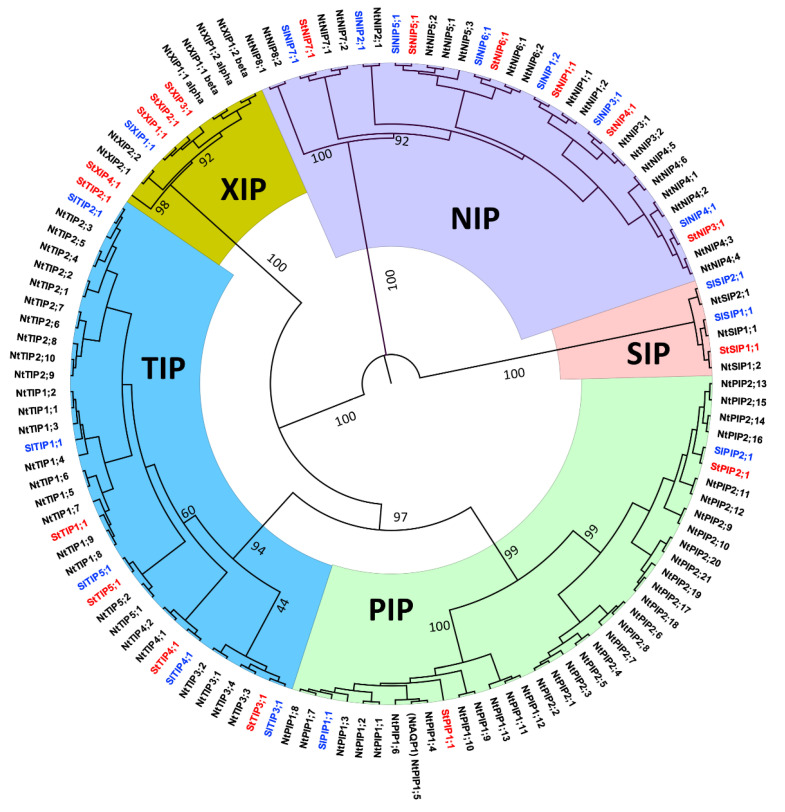
Phylogenetic relationships among *Nicotiana tabacum*, *Solanum tuberosum*, and *Solanum lycopersium* AQPs. For this analysis, 35 selected subgroup representative StAQPs and SlAQPs were aligned with all NtAQPs using the Clustal Omega server (http://www.ebi.ac.uk/Tools/msa/Clustal Omega/) and a phylogenetic tree was constructed using Maximum Likelihood method based on the JTT matrix-based model with 1000 bootstraps. AQPs clustered into five different subfamilies (PIPs, TIPs, NIPs, SIPs, and XIPs). Each AQP subfamily is shown with a specific background color. NtAQPs are indicated in black; StAQPs and SlAQPs are in red and blue, respectively.

**Figure 2:**
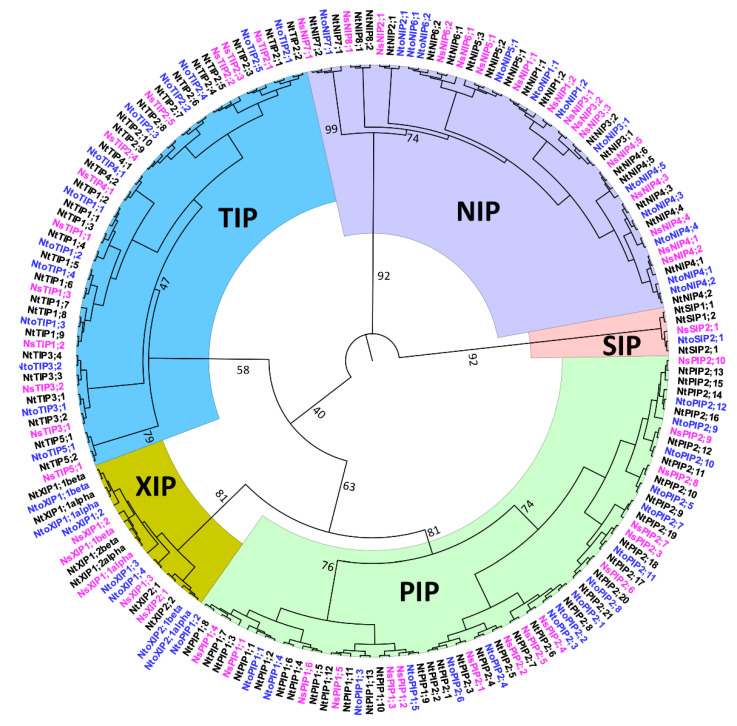
Phylogenetic relationships among *N. tabacum (Nt)* AQPs and its two ancestors, *N. sylvestris (Ns)* and *N. tomentosiformis (Nto)* AQPs. The deduced amino acid sequences of NtAQPs, NtoAQPs, and NsAQPs were aligned using the Clustal Omega server (http://www.ebi.ac.uk/Tools/msa/Clustal Omega/) and a phylogenetic tree was constructed using Maximum Likelihood method based on the JTT matrix-based model with 1000 bootstraps. The NtAQPs clustered into five different subfamilies (PIPs, TIPs, NIPs, SIPs and XIPs), with the corresponding NtoAQP and NsAQP subfamilies. Each AQP subfamily is shown with a specific background color. NtAQPs are indicated in black, NtoAQPs are in blue, and NsAQPs are in magenta.

**Figure 3:**
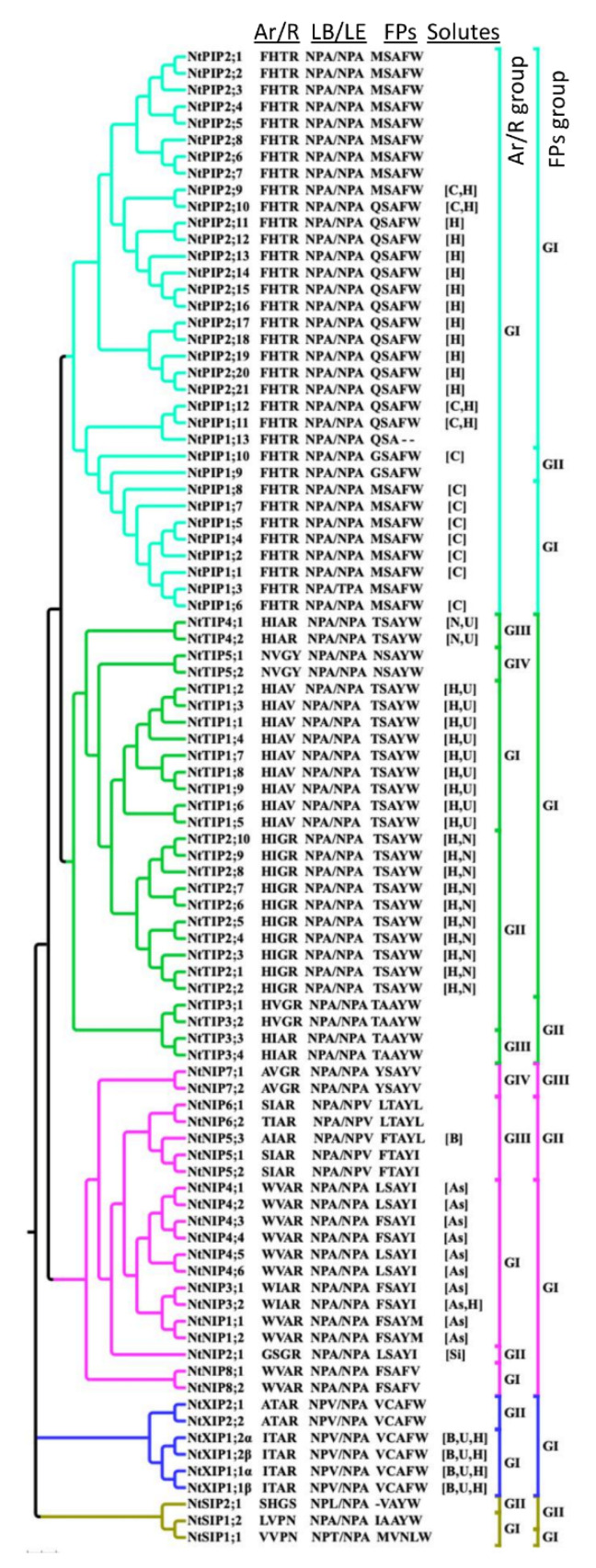
Grouping of *N. tabacum* PIPs, TIPs, NIPs, SIPs, and XIPs based on the ar/R and FPs. The phylogenetic tree was generated as described in Figure 1. The residues in the ar/R selectivity filter and the FPs were identified from the multiple sequence alignment, shown in Figures S1–S5. The ar/R and FP groupings within each subfamily were done based on the corresponding amino acid compositions, which are indicated on the right side of the phylogenetic tree. The solutes predicted, based on substrate specific signature sequences to be transported, are mentioned in square brackets. As, B, C, H, N, Si, Sb, and U indicate arsenic, boron, CO_2_, H_2_O_2_, ammonia, silicon, antimony, and urea, respectively.

This represents the greatest *AQP* gene number in a Solanaceae plant genome. We identified 49 *AQP* genes encoding 51 and 50 full-length proteins in two *N. tabacum* ancestors, namely *N. tomentosiformis* and *N. sylvestris*, respectively, as shown in Table S1. The phylogenetic protein analysis showed that NtAQPs cluster into five subfamilies (PIPs, TIPs, NIPs, SIPs, and XIPs) similar to NtoAQPs, NsAQPs, and SlAQP and StAQP, as shown in Figure 1, Figure 2 and Figure 3. NtAQPs nomenclature was done from protein sequence comparison with the known SlAQP and StAQP, as shown in Figure 1. Sequences belonging to hybrid intrinsic proteins (HIPs) and GlpF-like intrinsic proteins (GIPs) reported in the non-vascular moss *Physcomitrella patens* [14] were not found. In *N. tabacum*, we identified 34 PIPs, 27 TIPs, 20 NIPs, 3 SIPs, and 6 XIPs, including two splice variants. Figure 1 shows that the PIPs cluster either into the PIP1 or PIP2 groups, and the NtTIPs into five groups (TIP1 to TIP5), similar to the potato and tomato TIPs [3,7]. Eight NIP groups were found in *N. tabacum*, contrary to the seven groups in *Arabidopsis* and soybean [5,11], and three to four NIP groups in poplar, rice, and maize [6,10,12]. Similar to *Arabidopsis*, rice, maize, poplar, and soybean, *N. tabacum* had two SIP groups, namely SIP1 and SIP2s, with two and one isoforms, respectively. Two XIP subgroups were observed in *N. tabacum*, and four XIP subgroups in potato [3].

Subcellular localization prediction was conducted using WoLF PSORT software, and the results were as follows: NtPIPs–plasma membrane (PM) and chloroplast, as shown in Table 1; TIPs–vacuole and PM; NIPs–PM and vacuole; SIPs–PM (SIP2;1 in both the PM and chloroplast); XIPs–PM. These localizations are just predictions and need to be experimentally demonstrated. Part of the predictions are in agreement with the data reported in the literature, but many differences are also observed. For instance, plant PIP2s are not found in the chloroplasts, TIPs are mostly located in the vacuole (and not in the PM, as predicted for many NtTIPs), and NIPs were not identified in the vacuole. SIPs were localized in the PM and/or the ER in Arabidopsis and maize [40] (Lebrun and Chaumont, unpublished data), but never in the chloroplast. The amino acid number, calculated molecular weight (MW), and isoelectric point (pI) of NtAQP homologs are shown in Table 1.

Like their counterparts in other plant species, all PIPs, TIPs, NIP1s, NIP2s, NIP3s, NIP4s, NIP7s, and NIP8s from *N. tabacum,* have two conserved NPA motifs in loops B and E, as shown in Figure 3 and Figures S1–S5. NIP5s and NIP6s have unusual NPA motifs, in which the alanine in loop E is substituted by a valine, and have a characteristic arginine-rich C-terminus, as shown in Figure 3 and Figure S3. In *N. tabacum* SIPs, the alanine in the first NPA motif is substitued by either a threonine (SIP1;1) or a leucine (SIP2s) residue, as shown in Figure 3 and Figure S4. All the SIPs have the conserved NPA motif in loop E with a unique characteristic lysine-rich C-terminus, as shown in Figure S4, which contains an ER retention signal [1,41] (Lebrun and Chaumont, unpublished). In the *N. tabacum* genome, there are four *XIP* genes, including *NtXIP1;1* and *NtXIP1;2*, which encode two splice variants (α and β) [15]. In *N. tabacum* XIPs in the first NPA motif (loop B), alanine is substituted by a valine residue, as shown in Figure 3 and Figure S5.

### 2.2. NtAQP Gene Structures

**Figure 4:**
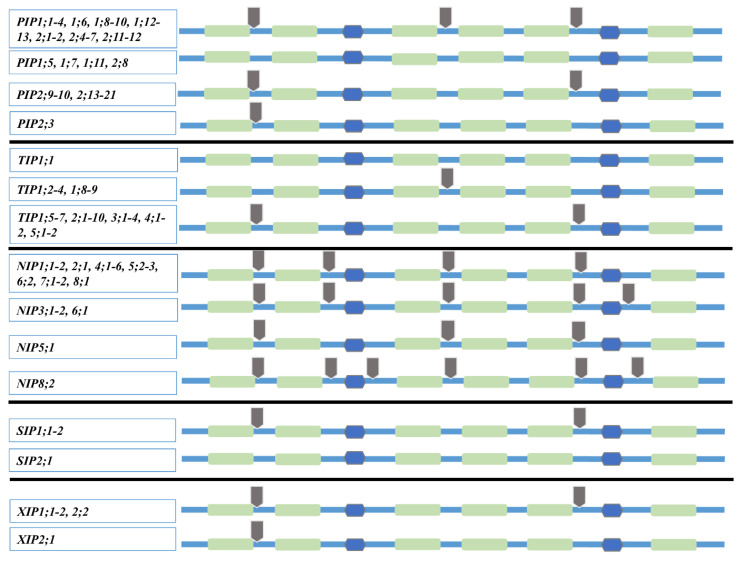
*NtAQP* genes and 2-D protein structure. Introns in the *NtAQP* genes are indicated by gray arrows. The six TM regions are shown in light green bars, and loops B and E are shown in blue hexagons.

The *N. tabacum AQP* genomic sequences were analyzed for introns and exons, as shown in Figure 4 and Figure S6. Apart from a few inconsistencies, the number and position of introns are conserved within each *AQP* subfamily. *NtPIP* genes have two or three introns, except for *NtPIP2;3*, which has a single intron, and *NtPIP1;5*, *NtPIP1;7*, *NtPIP1;11*, and *NtPIP2;8*, which have no introns, as shown in Figure 4. Among them, *NtPIP2;2* has a very long intron (~15 kb), as shown in Figure S6. The *NtTIP* subfamily exhibits relatively stable gene structure in comparison with other subfamilies. The majority of them have two introns except for *TIP1;2–4* and *TIP1;8–9*, which have a single intron and *NtTIP1;1* with no intron, as shown in Figure 4. The majority of *NtNIPs* have four introns with variable intron-exon organization, as shown in Figure 4 and Figure S6. *NtNIP5;1* has three introns, and *NtNIP3s* and *NtNIP6;1* have five introns, while *NtNIP8;2* possesses a unique gene structure with six introns (the greatest number of introns in an *AQP* gene), one of which is 10 kb long, as shown in Figure S6. The *NtSIP* genes have two introns, except for *NtSIP2;1,* which has no intron. The *NtXIPs* gene structure was very conserved with two introns, except for *NtXIP2;1,* which has a single intron, as shown in Figure 4.

### 2.3. Analysis of NtAQPs Ar/R Selectivity Filter and Froger’s Position

We identified the four amino acid residues at the ar/R selectivity filter and the five residues in the FPs using sequence alignments, and used them to group the NtAQPs based on the amino acid residue properties and to compare these groups with those of other species, such as tomato and potato, as shown in Figure 3 [3,7,9]. In addition, all NtAQPs were subjected to the ScanProsite tool (http://prosite.expasy.org/scanprosite/), to identify the substrate specificity-determining positions (SDPs) based on the ar/R, FP, and NPA motifs, and thereby the predicted substrate(s) of each isoform, as shown in Table 2, Figure 3, and Table S2. Water is considered as the universal substrate for AQPs, even though some isoforms were shown not to facilitate its diffusion through the membrane [15].

The ar/R selectivity filter in all the NtPIPs is composed of F, H, T, and R residues in TM2, TM5, LE1, and LE2, respectively, and is identical to the ar/R filter found in all the plant PIPs, as shown in Figure 3. According to the residues located at the P1 of FPs, M or Q (G), NtPIPs cluster into two groups, I and II, as shown in Figure 3. Twelve PIPs (mainly PIP1s) are predicted CO_2_ channels and thirteen PIPs (mainly PIP2s) are predicted H_2_O_2_ channels, as shown in Figure 3. Based on the ar/R filter, the NtTIPs cluster into four groups (I, II, III, and IV), as shown in Figure 3. The P3–P5 positions in FPs of all NtTIPs are conserved and consist of A, Y, and W residues, respectively, as shown in Figure 3. Based on the disparities in P1 and P2 positions, all TIPs could be divided into two groups. TIP1s and TIP2s are predicted H_2_O_2_ channels, and TIP1s and TIP4s are predicted urea channels, as shown in Figure 3. TIP2s and TIP4s are also predicted as NH_3_ channels, which is in agreement with experimental evidence in other species [18,42]. Based on the ar/R selectivity filters, all NtNIPs are divided into four different groups, as shown in Figure 3. On the other hand, based on the FPs, NtNIPs cluster into three groups, as shown in Figure 3, such as potato and tomato, but unlike other plants (Arabidopsis, maize, etc.) [3,6,7,11]. Our analysis predicted that the As transporters are only distributed among the NtNIPs (10 NIPs belonging to Group I, based on the ar/R filter and FPs), as shown in Figure 3. NIP2;1, NIP5;1, and NIP3;2 are predicted as Si, B, and H_2_O_2_ channels, respectively. The NtSIPs are grouped into two groups based on both the ar/R selectivity filter and FPs, as shown in Figure 3. Very few studies have examined the channel specificity of SIPs. Two SIPs from Arabidopsis showed some water channel activity when expressed in yeast [40]. The NtXIPs are clustered into two groups based on the ar/R selectivity filter. However, based on FPs, all NtXIPs were grouped in a single group, as shown in Figure 3. XIP1;1 and XIP1;2 are predicted as B, urea, and H_2_O_2_ channels, as shown in Figure 3. The specificity and function of NtXIP1;1, including its splice variant, were studied in detail and were shown to facilitate the diffusion of B, H_2_O_2_, NH_3_, and urea, but not water [15,43].

### 2.4. Expression of NtAQP Genes in Roots, Leaves, and Flowers as well as BY-2 Suspension Cells

**Figure 5:**
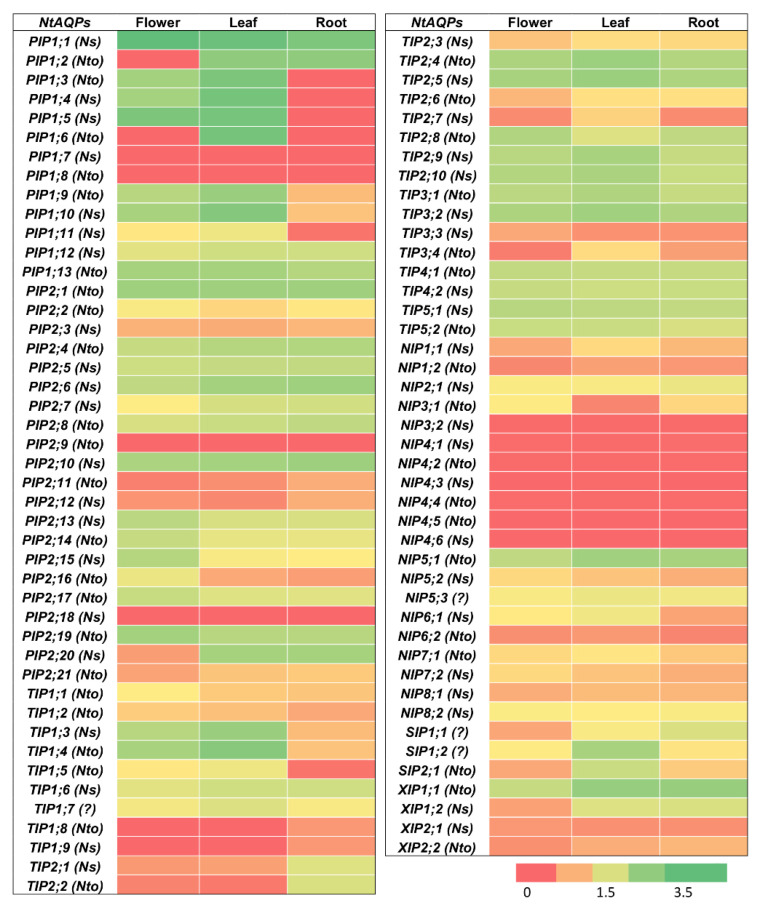
Expression analyses of 88 *NtAQP* genes in root, leaf, and flower. Color scale represents logarithmic FPKM values, where green indicates high expression and red indicates no or very low expression. *Ns* and *Nto* in parentheses indicate that corresponding *NtAQP* gene evolved from *N. sylvestris (Ns)* or *N. tomentosiformis (Nto).* Question mark (?) indicates that *NtAQP* gene origin (*N. sylvestris* or *N. tomentosiformis*) was not identified.

The heatmap based on FPKM values shows the *NtAQPs* transcript levels in roots, leaves, and flowers, as shown in Figure 5. Among the 88 *NtAQPs* genes, 73, 75, and 71 are expressed in mature flowers, leaves, and roots, respectively, and 68 genes are ubiquitously expressed in all analyzed organs. *PIPs* are expressed in flowers, leaves, and roots but differently according to the isoforms. A greater number of *NtPIP1* genes are expressed in flowers and leaves than in roots—*NtPIP1;1* and *NtPIP1;10* being the most expressed isoforms in flowers and leaves, respectively, and *NtPIP1;3–8* and *NtPIP1;11* not being expressed in roots. A decreased amount of *NtPIP2* transcripts is generally observed, but all *NtPIP2s* are expressed in the three organs with the exception of *NtPIP2;9* and *NtPIP2;18*, which are not expressed or are expressed very little, as shown in Figure 5*. NtTIP* gene expression levels are often greater in the leaves compared with the other organs, even if a greater number of *NtTIP* genes are expressed in roots, as shown in Figure 5. Among the 20 *NtNIP* genes, seven (*NtNIP3;2* and all the *NtNIP4s*) are not or very lowly expressed in the three organs in the tested conditions. The other *NtNIP* genes are relatively less expressed compared to the other *AQP* subfamily members, as shown in Figure 5. All *NtSIP* genes were ubiquitously expressed in flowers, leaves, and roots, *NtSIP1;2* being the most expressed *NtSIP* in the leaf, as shown in Figure 5. Finally, *NtXIP1;1* was the most expressed *NtXIP* in the three organs with the expression of the others being very decreased. 

**Figure 6:**
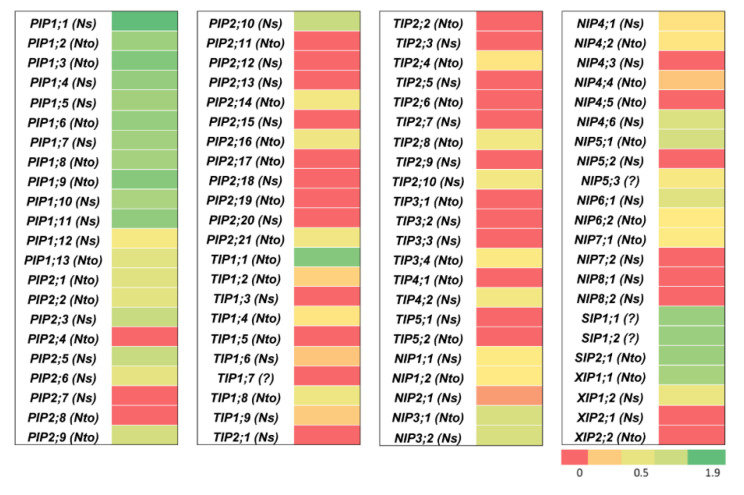
Expression analyses of 88 *NtAQP* genes in *N. tabacum* BY-2 cells. Color scale represents logarithmic FPKM values, where green indicates high expression and red indicates no expression or very low expression. *Ns* and *Nto* in parentheses indicate that corresponding *NtAQP* gene evolved from *N. sylvestris (Ns)* or *N. tomentosiformis (Nto).* Question mark (?) indicates that *NtAQP* gene origin (*N. sylvestris* or *N. tomentosiformis*) was not identified.

*N. tabacum* BY-2 suspension cells are widely used to study different physiological processes, the role of specific proteins, or as a heterologous expression system to produce high value pharmaceutical antigens or antibodies [44,45,46,47,48]. We determined which *AQP* genes are expressed in those cells that grow in suspension in an aqueous environment. RNA from wild-type BY-2 cells was extracted and RNA-seq data analyzed for the expression of the 88 *NtAQP* genes. The heatmap based on FPKM values is shown in Figure 6. mRNA of 53 *NtAQP* genes were detected in BY-2 cells growing in a standard MS medium. The most expressed *NtAQP* genes were 11 *PIP1s*, *TIP1;1*, the three *SIPs,* and *XIP1;1*.

## 3. Discussion

By screening the *N. tabacum* genome databases, we identified 88 complete *AQP* genes, almost twice the number of *AQP* genes identified in tomato and potato [3,7]. The number of *AQP* homologs always varies between plant species, the dicot plant genomes usually encoding more homologs than the monocot plants, except for the 68 full-length *AQP* genes found in *P. virgatum*, a polyploid monocot species [9]. The great number of *AQP* genes in the *N. tabacum* genome arose from an allotetraploidization event that occurred about 200,000 years ago [36,38] between *N. tomentosiformis* and *N. sylvestris*, which each have 49 *AQP* genes. The difference between the identified gene number in *N. tabacum* (88) and the sum of the *N. tomentosiformis* and *N. sylvestris AQP* genes (98) suggests that some were lost after the polyploidization event. In addition, we also could not exclude the recent local duplication events in each species, as deduced by the protein phylogenetic tree, shown in Figure 2, in which two very close isoforms from the same species are found on the same branch (i.e., NtPIP2;1 and 2;2, NtoPIP2;2 and 2;3, NsNIP3;1 and 3;2, NtoNIP6;1 and 6;2, etc.). Models have been proposed to explain duplicated gene fate: pseudogenization, sub-functionalization, and neo-functionalization [49]. Redundancy also allows one of the copies to accumulate mutations without affecting plant fitness, and new allelic variants or changes in the gene expression pattern can be observed [50]. While activity determination of the duplicated isoforms would be required to determine a sub- or neo-functionalization, changes in expression patterns can be deduced from the rough *NtAQP* expression data analysis. For instance, the duplicated *NtPIP2;1* and *NtPIP2;2* showed different expression levels, which can be organ dependent. 

We identified five subfamilies (PIP, TIP, NIP, SIP, and XIP) among the three *Nicotiana* species, similar to most other dicots, except for Brassicaceae and monocots, which have no XIP subfamily [15]. Several *N. tabacum* AQPs have been characterized [51,52,53,54,55], and some became paradigms in the plant AQP community [21,22]. NtAQP1, corresponding to NtPIP1;5 in our study, is a PIP1 protein located both in the plasma membrane and the chloroplast envelope, which exhibits water and CO_2_ channel permeability [21]. This discovery highlighted the important diverse roles of AQPs in plant physiology and, more particularly, in photosynthesis, through their contribution in facilitating CO_2_ membrane diffusion [28]. More recently, the membrane diffusion of another gas, O_2_, was reported to be facilitated by NtPIP1;3 when expressed in yeast, and an increased *NtPIP1;3* transcript level was measured in *N. tabacum* roots after a seven day hypoxia treatment [22], suggesting a potential new physiological role of plant AQPs in O_2_ membrane permeability. NtXIP1s are the first plant XIP isoforms that have been functionally characterized [15]. NtXIP1;1 is located in the plasma membrane and is shown in a functional assay in heterologous systems to facilitate the membrane diffusion of H_2_O_2_, glycerol, boron, and urea, but not water [15]. NtXIP1;1 overexpression in *N. tabacum* results in disturbed boron tissue distribution, leading to boron deficient phenotypes in meristems and young leaves [43]. Interestingly, the *NtXIP1;1* gene contains a sequence motif in the first intron that initiates an RNA-processing mechanism that results in two splice variants (α and β), resulting in two amino acid residue differences [15]. We also identified XIP spliced variants for *NtXIP1;2*, *NtoXIP1;1*, *NsXIP1;1*, and *NtoXIP2;1* isoforms, and also XIPs from *S. tuberosum* and *S. lycopersicum* [15], indicating a conservation of this genomic feature in the Solanaceae family.

To elucidate the substrate specificity of NtAQPs, different signature sequences, including SDPs, NPA motifs, ar/R filter, and FPs were identified, as shown in Figure 3 and Table 2. From this multiple analysis, a majority of PIP1s and PIP2s were predicted to facilitate CO_2_ and H_2_O_2_ diffusion, respectively, in addition to water, as shown in Figure 3. This was confirmed in functional assays performed for NtPIP1;5 (NtAQP1) and NtPIP2;1 [21,53,54]. Most TIPs have similar NPA and FPs, suggesting that differences in their substrate transport selectivity might be regulated by the ar/R filter residues. Based on this ar/R filter, Group I and Group II TIPs have a wider pore aperture, which might facilitate the diffusion of relatively larger substrates than water, such as urea, ammonia, and H_2_O_2_ [56,57,58]. NtTIP4;1 (NtTIPa) was indeed shown to be permeable to water and urea, but also glycerol [51]. NIPs are most diverse in their NPA motifs, ar/R filter, and FPs, suggesting various substrate transport selectivities for these subfamily members and putatively important physiological roles. NIPs are also known to facilitate the transport of metalloids, such as arsenic and boron, as shown in Figure 3. NtXIPs are predicted to transport H_2_O_2_, boric acid, and urea, and were confirmed in transport assays performed with NtXIP1;1 [15,43]. In addition, NtXIP1;1 is not a water channel but is able to facilitate glycerol diffusion [15]. Finally, limited information is available for plant SIP specificity. Water channel activity was determined for AtSIP1s, unlike for AtSIP2;1 [40]. This global substrate specificity study, based on prediction is, however, to be taken with caution, as a single amino acid change, even in the transmembrane domains, could affect the channel characteristic or conformation [59]. Therefore functional assays in heterologous or homologous systems will have to be carried out when analyzing the functional role of specific NtAQP. 

As expected, *NtAQP* transcript levels are dependent on the plant organs, but it is quite surprising to observe that 68 of 88 *AQP* genes are ubiquitously expressed in roots, young leaves, and flowers. *PIP* and *TIP* transcripts are relatively more abundant than other subfamily mRNAs, as shown in Figure 5. Considering that the main role of these isoforms is the water facilitated permeation through plasma and vacuolar membranes, this observation confirms their primordial role in water movement through plant tissues, in cell expansion, and cell water homeostasis [24,60]. The *NIP* expression level is low, except for *NtNIP5;1*, but due to their metalloid substrate specificity, a more restricted tissue/cell expression pattern in specific physiological conditions might be expected [43,61,62]. mRNA of 53 *NtAQP* genes were also detected in BY-2 suspension cells growing in a standard MS medium, even if the relative expression level between them was different to what was observed in plant organs. This could be due to the dedifferentiated nature of those cells and/or the specific cell environment of the culture medium. The most expressed *NtAQP* genes in BY-2 cells are 11 *NtPIP1s*, *NtTIP1;1*, the three *NtSIPs*, and *NtXIP1;1*. High *NtPIP* gene expression was also reported in maize Black Mexican Sweet (BMS) suspension cells [63], but in this case, the two most expressed genes belonged to the PIP2 group. Plant PIP1s physically interact with PIP2s within heterotetramers, leading to PIP1 relocalization from the endoplasmic reticulum to the plasma membrane [59,64]. We might wonder whether PIP2 abundance in BY-2 cells is sufficient to bring all PIP1s to the plasma membrane. The increased PIP expression in suspension cells suggests that they are important in controlling membrane water permeability during suspension cell growth. In fact, *PIP* expression varies according to BMS cell growth stages, and this is correlated with greater cell water permeability, measured at the end of the log phase and stationary phase [63]. This might be dependent on variations in the medium composition and/or internal osmotic pressure. *PIP* and *TIP* gene expression in BY-2 suspension cells might also be involved in the control of cell expansion. Cauliflower BobTIP26–1 overexpression in suspension cells (*N. tabacum* cv. Wisconsin 38) increases the cell volume [65], cell enlargement being mostly accounted by vacuole swelling. The quite high expression of *NtSIPs* is also intriguing, knowing that SIPs are mostly expressed in the endoplasmic reticulum and their function is still unknown. *ZmSIP1;2* is also expressed in BMS suspension cells, and its expression is not dependent on the growth stage [63]. Suspension cells might be a promising model to investigate the physiological role at the cell level as well as the biochemical properties of this AQP subfamily. Actually, BY-2 suspension cells represent very useful tools to study AQP function, localization regulation, substrate specificity, and structure, as the cells are easily transformed by *Agrobacterium tumefaciens* or biolistics, and great cell amounts could be obtained for protein purification and reconstitution [66].

In this comprehensive analysis, we identified a highly diverse AQP gene family in *N. tabacum* as well as in its two ancestors, *N. tomentosiformis* and *N. sylvestris*. The signature sequence for substrate selectivity and the possible biological function of NtAQPs were predicted. The transcriptomic data of *N. tabacum* and BY-2 suspension cells represent an excellent resource to guide further analysis of the function of any selected AQP isoform. 

## 4. Materials and Methods

### 4.1. Identification and Sequence Analysis of NtAQPs

The genomes of *N. tabacum*, *N. tomentosiformis*, and *N. sylvestris* available at the Sol Genomics Network (https://solgenomics.net/organism/Nicotiana_tabacum/genome), were searched for AQPs using BLASTp (http//http://www.ncbi.nlm.nih.gov/blast/Blast.cgi?PAGE = Proteins) tools with the protein sequences of 47 AQPs from *S. lycopersium* (tomato) and 41 AQPs from *S. tuberosum* (potato) as queries. Every sequence from each species was individually compared with functional annotations by browsing the *N. tabacum* databases.

### 4.2. Phylogenetic Analysis of N. Tabacum AQPs (NtAQPs)

NtAQPs amino acid sequences were separately aligned with *S. lycopersium* AQPs (SlAQPs) and *S. tuberosum* AQPs (StAQPs) using the Clustal Omega program (https://www.ebi.ac.uk/Tools/msa/clustalo/) and a phylogenetic tree was built using Molecular Evolution Genetic Analysis (MEGA), version 7.0 [67]. The phylogenetic analysis was conducted using the Maximum Likelihood method, based on the Jones–Taylor–Thornton (JTT) matrix-based model with 1000 bootstraps. The identified NtAQPs were classified into different subfamilies according to the phylogenetic relationships with SlAQPs and StAQPs.

### 4.3. Identification of NtAQP Gene Structure and Transmembrane Helices

Gene structures were determined by the GSDS 2.0 software (http://gsds.cbi.pku.edu.cn/) using the *NtAQP* gene and CDS sequences as input. The TM α-helices were predicted by TMpred (http://www.ch.embnet.org/software/TMPRED_form.html) and SOSUI (http://bp.nuap.nagoya-u.ac.jp/sosui/).

### 4.4. Prediction of Subcellular Localization

The subcellular localization of NtAQPs was predicted by using the WoLF PSORT (http://wolfpsort.org/), TargetP (www.cbs.dtu.dk/Services/TargetP), Cello prediction system (http://cello.life.netu.edu.tw/), and MultiLoc2 (www.abi.inf.uni-tuebingen.de/Services/MultiLoc2) tools. 

### 4.5. Identification of Substrate Specificity Determining Positions (SDPs)

The aligned NtAQP sequences were searched manually for SDPs by following the prediction explained previously [9,17] and clustered into different functional groups. The functional group sequences were aligned using Clustal Omega (https://www.ebi.ac.uk/Tools/msa/clustalo/).

### 4.6. Expression Profile of NtAQP Genes

Transcript levels as FPKM (Fragments per Kilobase of Transcript per Million Mapped Reads) values of *NtAQP* genes in different organs (mature flowers, leaves and roots) were obtained from the Gene Expression Omnibus (GEO) repository and GenBank Sequence Read Archive (SRA) under the accession code SRP029183 (SRX338104: *N. tabacum* TN90 root; SRX338101: *N. tabacum* TN90 leaf; SRX495520: *N. tabacum* TN90 mature flower). Three biological replicates were obtained from each organ. The FPKM values of the respective *NtAQP* genes were extracted from the databases and transformed into logarithmic (log_10_) values to generate the heatmap. A heatmap showing the logarithmic *NtAQP*s transcript levels in root, leaf, and flower was generated using Microsoft Excel conditional formatting, based on the normalized FPKM values. In our analysis, a logarithmic FPKM value > 0 was used as a threshold to consider whether a gene is expressed.

### 4.7. RNA-Seq Experiment

*N. tabacum* cv. BY-2 suspension cells were grown in the dark at 25 °C with agitation on a rotary shaker (90 rpm) in liquid MS medium (4.4 g/L Murashige and Skoog salts (MP BIOMEDICALS, Solon, OH), 30 g/L sucrose, 0.2 g/L KH_2_PO_4_, 2.5 mg/L thiamine, 50 mg/mL myo-inositol, and 0.2 mg/L 2,4-D, pH 5.8 (KOH)). Cultures were grown in 50 mL of medium in a 250 mL Erlenmeyer flask and a 5% inoculum was transferred each week into fresh medium. BY-2 cells (100 mg) were collected three days after inoculation (exponential phase) and the total RNA was extracted from three biological replicates and sent to the Macrogen Company, which performed the library preparation, RNA sequencing, and data analysis. For the library preparation, the mRNA was purified from total RNA and transformed into a template molecule library, appropriate for subsequent cluster generation using the Illumina^®^ TruSeq™ RNA Sample Preparation Kit. The first step in the workflow encompassed purifying the poly-A-containing mRNA molecules using poly-T oligo-attached magnetic beads. After purification, the mRNA was split into small pieces using divalent cations under high temperature. The cleaved RNA fragments were copied into first strand cDNA using reverse transcriptase and random primers. This was followed by the second strand cDNA synthesis using DNA polymerase I and RNase H. These cDNA fragments then went through an end repair process, the addition of a single “A” base, and then the ligation of adapters. Finally, the products were purified and enriched with PCR to generate the final cDNA library. The library was then submitted for paired-end 2 × 100 bp sequencing in Illumina HiSeq2000. Sequencing data were analyzed through the Trinity pipeline, which permitted de novo transcriptome reconstruction. The transcript abundances were calculated using RSEM (1.2.15) software [68]. Blast-X (https://blast.ncbi.nlm.nih.gov/Blast.cgi?PROGRAM = blastx&PAGE_TYPE = BlastSearch&LINK_LOC = blasthome) was used to compare the six-frame translation products of a nucleotide query sequence against a protein sequence database (go_v20150407). Finally, the FPKM values for the respective *AQP* genes were identified from the annotated BY-2 cell transcriptomic data. A heatmap was generated based on the transformed logarithmic (log_10_) FPKM values. Similar to organ specific expression data, FPKM values > 0 were used as a threshold to consider whether a gene is expressed.

## Figures and Tables

**Table 1:** Aquaporin genes in the *N. tabacum* genome.

Gene Name	Accession Number	IP^1^/MW (kDa)	Amino Acid Number	Predicted Subcellular Localization^2^
*NtPIP1;1*	NP_001313131.1	8.83/30.70	286	PM, C
*NtPIP1;2*	XP_016508253.1	8.30/30.76	285	PM
*NtPIP1;3*	AAB04757.1	9.08/30.58	287	PM
*NtPIP1;4*	NP_001312189.1	8.30/30.80	287	PM
*NtPIP1;5*	CAA04750.1	8.29/30.82	287	PM
*NtPIP1;6*	XP_016476491.1	8.99/30.90	287	PM
*NtPIP1;7*	NP_001312824.1	8.61/30.84	287	PM
*NtPIP1;8*	NP_001312222.1	8.83/30.82	286	PM
*NtPIP1;9*	NP_001312921.1	8.96/30.49	284	PM
*NtPIP1;10*	XP_016458231.1	9.10/30.74	288	PM
*NtPIP1;11*	XP_016515710.1	8.23/27.31	254	PM
*NtPIP1;12*	NP_001312721.1	8.99/30.77	287	PM
*NtPIP1;13*	XP_016510215.1	9.00/30.64	285	PM
*NtPIP2;1*	AAL33586.1	9.05/30.49	268	PM, C
*NtPIP2;2*	NP_001313091.1	9.05/30.47	268	PM
*NtPIP2;3*	NP_001312414.1	9.04/30.48	268	PM
*NtPIP2;4*	NP_001312350.1	8.87/30.41	283	PM
*NtPIP2;5*	NP_001312874.1	8.89/30.39	283	PM
*NtPIP2;6*	XP_016477641.1	9.02/28.49	284	PM
*NtPIP2;7*	NP_001313061.1	8.98/28.63	284	PM
*NtPIP2;8*	XP_016476355.1	9.17/28.49	284	PM, C
*NtPIP2;9*	NP_001312511.1	8.84/30.37	283	PM
*NtPIP2;10*	XP_016494749.1	8.63/30.32	283	PM
*NtPIP2;11*	NP_001311701.1	8.19/30.49	285	PM
*NtPIP2;12*	NP_001312276.1	7.62/30.48	285	PM
*NtPIP2;13*	NP_001312334.1	6.94/31.23	291	PM
*NtPIP2;* *14*	XP_016486700.1	6.94/31.21	291	PM
*NtPIP2;* *15*	NP_001312333.1	7.62/30.26	283	PM
*NtPIP2;16*	XP_016513533.1	7.62/30.30	283	PM
*NtPIP2;17*	NP_001312464.1	8.21/30.73	287	PM
*NtPIP2;18*	NP_001313066.1	8.20/30.78	287	PM
*NtPIP2;19*	NP_001313208.1	7.04/30.16	283	PM
*NtPIP2;* *20*	NP_001311719.1	7.04/30.73	287	PM
*NtPIP2;* *21*	NP_001311765.1	7.69/30.68	287	PM
*NtTIP1;1*	BAF95576.1	5.55/25.79	252	PM
*NtTIP1;2*	NP_001312131.1	5.70/25.80	252	PM, V
*NtTIP1;3*	NP_001312871.1	5.70/25.73	248	PM
*NtTIP1;4*	XP_016513281.1	5.91/26.19	248	PM
*NtTIP1;5*	XP_016501711.1	5.37/25.91	248	PM
*NtTIP1;6*	XP_016487055.1	5.37/25.90	251	PM
*NtTIP1;7*	XP_016471957.1	6.04/25.56	251	PM
*NtTIP1;8*	XP_016495978.1	5.62/25.12	251	PM, V
*NtTIP1;9*	XP_016450483.1	5.89/25.25	251	PM
*NtTIP2;1*	NP_001312646.1	5.35/24.94	248	PM, V
*NtTIP2;2*	XP_016495734.1	5.35/24.99	248	PM
*NtTIP2;3*	XP_016503582.1	6.00/25.07	248	PM
*NtTIP2;4*	XP_016480756.1	5.67/25.01	248	PM
*NtTIP2;5*	XP_016515893.1	5.67/25.02	248	PM
*NtTIP2;6*	XP_016445220.1	4.85/25.36	250	V
*NtTIP2;7*	XP_016481958.1	4.85/25.30	250	V
*NtTIP2;8*	NP_001312940.1	5.66/25.23	250	V
*NtTIP2;9*	XP_016481922.1	5.66/25.24	250	V
*NtTIP2;10*	P24422.2	5.32/25.22	250	V
*NtTIP3;1*	XP_016491554.1	7.07/27.62	260	PM
*NtTIP3;2*	XP_016491898.1	8.08/27.58	260	PM
*NtTIP3;3*	XP_016436583.1	7.07/27.41	259	PM
*NtTIP3;4*	XP_016500896.1	7.07/27.40	259	PM
*NtTIP4;1*	NP_001311953.1	5.79/25.96	247	V
*NtTIP4;2*	XP_016441470.1	5.79/25.98	247	V
*NtTIP5;1*	XP_016462485.1	7.78/25.63	250	PM
*NtTIP5;2*	XP_016485861.1	7.78/25.59	250	PM
*NtNIP1;1*	XP_016487110.1	9.08/30.67	288	PM
*NtNIP1;2*	XP_016445609.1	9.41/32.65	303	PM
*NtNIP2;1*	XP_016451246.1	8.96/30.49	286	PM
*NtNIP3;1*	XP_016460638.1	8.29/37.69	337	PM
*NtNIP3;2*	XP_016515586.1	8.29/37.91	347	PM
*NtNIP4;1*	XP_016486634.1	8.52/29.73	281	V
*NtNIP4;2*	XP_016455585.1	8.83/29.12	275	V
*NtNIP4;3*	XP_016491262.1	7.74/28.43	270	PM
*NtNIP4;4*	XP_016453373.1	6.89/28.67	271	PM
*NtNIP4;5*	XP_016456203.1	8.28/29.07	272	PM
*NtNIP4;6*	XP_016500017.1	7.69/29.16	272	PM
*NtNIP5;1*	XP_016470302.1	8.63/30.98	297	V
*NtNIP5;2*	NP_001312819.1	8.87/30.91	297	V
*NtNIP5;3*	XP_016493176.1	9.86/31.94	304	PM
*NtNIP6;1*	XP_016435920.1	8.73/34.50	331	V
*NtNIP6;2*	XP_016438237.1	8.66/32.35	313	PM
*NtNIP7;1*	XP_016509644.1	7.71/29.58	280	PM
*NtNIP7;2*	XP_016496646.1	7.78/31.18	293	PM
*NtNIP8;1*	XP_016468207.1	8.78/29.88	277	V
*NtNIP8;2*	XP_016451938.1	9.22/34.00	314	PM
*NtSIP1;1*	XP_016439604.1	9.22/25.06	238	PM
*NtSIP1;2*	XP_016492107.1	9.55/25.94	242	PM
*NtSIP2;1*	XP_016496337.1	10.01/26.45	240	PM, C
*NtXIP1;1α*	NP_001312796	7.70/34.61	325	PM
*NtXIP1;1β*	Nitab4.5_0000956g0150.1	7.71/34.75	325	PM
*NtXIP1;2α*	XP_016446694	7.71/34.68	326	PM
*NtXIP1;2β*	Nitab4.5_0007293g0050.1	7.71/34.54	326	PM
*NtXIP2;1*	XP_016489264.1	6.05/33.40	313	PM
*NtXIP2;2*	XP_016488683	8.70/33.07	308	PM

^1^ IP = Isoelectric point. ^2^ PM: plasma membrane, C: chloroplast, V: vacuole.

**Table 2:** Substrate specificity determining positions (SDPs) in NtAQPs.

Substrates	Ar/R (H2-H5-LE1-LE2)	LB (NPA Region)	LE (NPA Region)	FPs (P1-P5)	Transporters Based on Those SDP Positions
**Bo**	[AGI][ISV][GA]R	SG[AG]H[ILM]NP[ASV][VLI][TS]	[GS][GA][SG]MNP[AV]R[STC][LF]G	[FIV][TC]A[YF][LFW]	NtNIP5;1, NtXIP1;1–2
**CO_2_**	FHTR	SGGHINPAVT	GTGINPARSLG	[MQ]SAFW	NtPIP1;1–2, NtPIP1;4–8, NtPIP1;10, NtPIP1;12–13, NtPIP2;9–10
**H_2_O_2_**	[HFWI][IHV][ATG][VR]	SG[GA]H[VLIF]NP[AV][VI][TS]	G[AGT][SG][MI]NP[AG][VR][ASC][FL]G	[TQFV][ASC]A[YF][WI]	NtPIP1;12–13, NtPIP2;9–21, NtTIP1;1–9, NtTIP2;1–10, NtNIP3;2, NtXIP1;1–2
**NH_3_**	[HW][IV][AG]R	SGGH[VLF]NPAVT	G[GA]SMNPARS[FL]G	[FT]SAY[LW]	NtTIP2;1–10, NtTIP4;1–2
**Si**	GSGR	SGAHMNPAVT	GGSMNPARTL[GA]	[IL]TAYF	NtNIP2;1
**U**	[HGANI][ISV][AG][RVC]	SG[GA]H[ILVM]NP[AV][VI][TS]	[GS][AG][SG]MNP[AV][RVC][TSC][LF]G	[MTLFVI][SATC]A[YF][WFL]	NtTIP1;1–9, NtTIP4;1–2, NtXIP1;1–2
**As**	[GAW][VSAI][GA][RV]	SG[AC]H[LIVMF]NP[AS][VI]T	[GS][GA]SMNP[AV]R[ST][LI][AG]	[LIFY][TS]AY[FILM]	NtNIP1;1–2, NtNIP3;1–2, NtNIP4;1–6
**Sb**	[AGT][IVSA][GA]R	SG[AC]H[LM]NP[SA][VIT][TS]	[GS][GA]SMNP[VA]R[TS]L[GA]	[FYIL][TS]AY[LMF]	-

Bo, Boron; H_2_O_2_, Hydrogen peroxide; CO_2_, Carbon dioxide; U, Urea; NH_3_, ammonia; As, arsenic; Sb, antimony; Si, silicon.

## Supplementary Materials

Supplementary Materials can be found at https://www.mdpi.com/1422-0067/21/13/4743/s1.

## Abbreviations

AQPs	Aquaporins
ar/R	Aromatic/arginine
As	Arsenic
B	Boron
BY-2	Bright Yellow-2
FPKM	Fragments per Kilobase of Transcript per Million Mapped Reads
FPs(P1–P5)	Froger’s positions
GEO	Gene Expression Omnibus
GIPs	GlpF-like intrinsic proteins
H_2_O_2_	Hydrogen peroxide
HIPs	Hybrid intrinsic proteins
JTT	Jones–Taylor–Thornton
LE	Loop E
MEGA	Molecular Evolution Genetic Analysis
MIPs	Major intrinsic proteins
MW	Molecular weight
NH_3_	Ammonia
NIPs	Nodulin-26-like intrinsic proteins
NPA	Asn-Pro-Alanine
pI	Isoelectric point
PIPs	Plasma membrane intrinsic proteins
Sb	Antimony
SDPs	Substrate specificity-determining positions
Si	Silicon
SIPs	Small basic intrinsic proteins
SRA	Sequence Read Archive
TIPs	Tonoplast intrinsic proteins
TM	Transmembrane
U	Urea
XIPs	Uncharacterized X intrinsic proteins

## References

[1] Gomes D., Agasse A., Thiébaud P., Delrot S., Gerós H., Chaumont F. (2009). Aquaporins are multifunctional water and solute transporters highly divergent in living organisms. Biochim. Biophys. Acta -Biomembr..

[2] Maurel C., Verdoucq L., Luu D.-T., Santoni V. (2008). Plant aquaporins: Membrane channels with multiple integrated functions. Annu. Rev. Plant Biol..

[3] Venkatesh J., Yu J.-W., Park S.W. (2013). Genome-wide analysis and expression profiling of the *Solanum tuberosum* aquaporins. Plant Physiol. Biochem..

[4] Bansal A., Sankararamakrishnan R. (2007). Homology modeling of major intrinsic proteins in rice, maize and Arabidopsis: Comparative analysis of transmembrane helix association and aromatic/arginine selectivity filters. BMC Struct. Biol..

[5] Zhang D.Y., Ali Z., Wang C.B., Xu L., Yi J.X., Xu Z.L., Liu X.Q., He X.L., Huang Y.H., Khan I.A. (2013). Genome-wide sequence characterization and expression analysis of major intrinsic proteins in soybean (*Glycine max* L.). PLoS ONE.

[6] Chaumont F., Barrieu F., Wojcik E., Chrispeels M.J., Jung R. (2001). Aquaporins constitute a large and highly divergent protein family in maize. Plant Physiol..

[7] Reuscher S., Akiyama M., Mori C., Aoki K., Shibata D., Shiratake K. (2013). Genome-wide identification and expression analysis of aquaporins in tomato. PLoS ONE.

[8] Park W., Scheffler B.E., Bauer P.J., Campbell B.T. (2010). Identification of the family of aquaporin genes and their expression in upland cotton (*Gossypium hirsutum* L.). BMC Plant Biol..

[9] Azad A.K., Ahmed J., Alum M.A., Hasan M.M., Ishikawa T., Sawa Y., Katsuhara M. (2016). Genome-wide characterization of major intrinsic proteins in four grass plants and their non-aqua transport selectivity profiles with comparative perspective. PLoS ONE.

[10] Gupta A.B., Sankararamakrishnan R. (2009). Genome-wide analysis of major intrinsic proteins in the tree plant *Populus trichocarpa*: Characterization of XIP subfamily of aquaporins from evolutionary perspective. BMC Plant Biol..

[11] Johanson U., Karlsson M., Johansson I., Gustavsson S., Sjövall S., Fraysse L., Weig A.R., Kjellbom P. (2001). The complete set of genes encoding major intrinsic proteins in Arabidopsis provides a framework for a new nomenclature for major intrinsic proteins in plants. Plant Physiol..

[12] Sakurai J., Ishikawa F., Yamaguchi T., Uemura M., Maeshima M. (2005). Identification of 33 rice aquaporin genes and analysis of their expression and function. Plant Cell Physiol..

[13] Ishibashi K., Hara S., Kondo S. (2009). Aquaporin water channels in mammals. Clin. Exp. Nephrol..

[14] Danielson J.Å., Johanson U. (2008). Unexpected complexity of the aquaporin gene family in the moss *Physcomitrella patens*. BMC Plant Biol..

[15] Bienert G.P., Bienert M.D., Jahn T.P., Boutry M., Chaumont F. (2011). Solanaceae XIPs are plasma membrane aquaporins that facilitate the transport of many uncharged substrates. Plant J..

[16] Hachez C., Chaumont F. (2010). Aquaporins: A family of highly regulated multifunctional channels. Adv. Exp. Med. Biol..

[17] Hove R.M., Bhave M. (2011). Plant aquaporins with non-aqua functions: Deciphering the signature sequences. Plant Mol. Biol..

[18] Di Giorgio J.P., Soto G., Alleva K., Jozefkowicz C., Amodeo G., Muschietti J.P., Ayub N.D. (2014). Prediction of aquaporin function by integrating evolutionary and functional analyses. J. Membr. Biol..

[19] Mukhopadhyay R., Bhattacharjee H., Rosen B.P. (2014). Aquaglyceroporins: Generalized metalloid channels. Biochim. Biophys. Acta -Gen. Subj..

[20] Jahn T.P., Møller A.L., Zeuthen T., Holm L.M., Klærke D.A., Mohsin B., Kühlbrandt W., Schjoerring J.K. (2004). Aquaporin homologues in plants and mammals transport ammonia. FEBS Lett..

[21] Uehlein N., Lovisolo C., Siefritz F., Kaldenhoff R. (2003). The tobacco aquaporin NtAQP1 is a membrane CO_2_ pore with physiological functions. Nature.

[22] Zwiazek J.J., Xu H., Tan X., Navarro-Ródenas A., Morte A. (2017). Significance of oxygen transport through aquaporins. Sci. Rep..

[23] Byrt C.S., Zhao M., Kourghi M., Bose J., Henderson S.W., Qiu J., Gilliham M., Schultz C., Schwarz M., Ramesh S.A. (2017). Non-selective cation channel activity of aquaporin AtPIP2;1 regulated by Ca^2+^ and pH. Plant Cell Environ..

[24] Chaumont F., Tyerman S.D. (2014). Aquaporins: Highly regulated channels controlling plant water relations. Plant Physiol..

[25] Azad A.K., Sawa Y., Ishikawa T., Shibata H. (2004). Phosphorylation of plasma membrane aquaporin regulates temperature-dependent opening of tulip petals. Plant Cell Physiol..

[26] Muto Y., Segami S., Hayashi H., Sakurai J., Murai-Hatano M., Hattori Y., Ashikari M., Maeshima M. (2011). Vacuolar proton pumps and aquaporins involved in rapid internode elongation of deep water rice. Biosci. Biotechnol. Biochem..

[27] Reinhardt H., Hachez C., Bienert M.D., Beebo A., Swarup K., Voß U., Bouhidel K., Frigerio L., Schjoerring J.K., Bennett M.J. (2016). Tonoplast aquaporins facilitate lateral root emergence. Plant Physiol..

[28] Uehlein N., Otto B., Hanson D.T., Fischer M., McDowell N., Kaldenhoff R. (2008). Function of *Nicotiana tabacum* aquaporins as chloroplast gas pores challenges the concept of membrane CO_2_ permeability. Plant Cell.

[29] Maurel C., Boursiac Y., Luu D.-T., Santoni V., Shahzad Z., Verdoucq L. (2015). Aquaporins in plants. Physiol. Rev..

[30] Chaumont F., Moshelion M., Daniels M.J. (2005). Regulation of plant aquaporin activity. Biol. Cell.

[31] Murata K., Mitsuoka K., Hirai T., Walz T., Agre P., Heymann J.B., Engel A., Fujiyoshi Y. (2000). Structural determinants of water permeation through aquaporin-1. Nature.

[32] Fu D., Libson A., Miercke L.J., Weitzman C., Nollert P., Krucinski J., Stroud R.M. (2000). Structure of a glycerol-conducting channel and the basis for its selectivity. Science.

[33] Sui H., Han B.-G., Lee J.K., Walian P., Jap B.K. (2001). Structural basis of water-specific transport through the AQP1 water channel. Nature.

[34] Heymann J.B., Engel A. (2000). Structural clues in the sequences of the aquaporins. J. Mol. Biol..

[35] Froger A., Thomas D., Delamarche C., Tallur B. (1998). Prediction of functional residues in water channels and related proteins. Protein Sci..

[36] Clarkson J.J., Dodsworth S., Chase M.W. (2017). Time-calibrated phylogenetic trees establish a lag between polyploidisation and diversification in *Nicotiana* (Solanaceae). Plant Syst. Evol..

[37] Sierro N., Battey J.N., Ouadi S., Bakaher N., Bovet L., Willig A., Goepfert S., Peitsch M.C., Ivanov N.V. (2014). The tobacco genome sequence and its comparison with those of tomato and potato. Nat. Commun..

[38] Sierro N., Battey J.N., Ouadi S., Bovet L., Goepfert S., Bakaher N., Peitsch M.C., Ivanov N.V. (2013). Reference genomes and transcriptomes of *Nicotiana sylvestris* and *Nicotiana tomentosiformis*. Genome Biol..

[39] Nagata T., Nemoto Y., Hasezawa S. (1992). Tobacco BY-2 cell line as the “HeLa” cell in the cell biology of higher plants. Int. Rev. Cytol..

[40] Ishikawa F., Suga S., Uemura T., Sato M.H., Maeshima M. (2005). Novel type aquaporin SIPs are mainly localized to the ER membrane and show cell-specific expression in *Arabidopsis thaliana*. FEBS Lett..

[41] Ishibashi K. (2006). Aquaporin subfamily with unusual NPA boxes. Biochim. Et Biophys. Acta (Bba)-Biomembr..

[42] Kirscht A., Kaptan S.S., Bienert G.P., Chaumont F., Nissen P., de Groot B.L., Kjellbom P., Gourdon P., Johanson U. (2016). Crystal structure of an ammonia-permeable aquaporin. PLOS Biol.

[43] Bienert M.D., Muries B., Crappe D., Chaumont F., Bienert G.P. (2019). Overexpression of X Intrinsic Protein 1; 1 in *Nicotiana tabacum* and Arabidopsis reduces boron allocation to shoot sink tissues. Plant Direct.

[44] Vasilev N., Grömping U., Lipperts A., Raven N., Fischer R., Schillberg S. (2013). Optimization of BY-2 cell suspension culture medium for the production of a human antibody using a combination of fractional factorial designs and the response surface method. Plant Biotechnol. J..

[45] Santos R.B., Abranches R., Fischer R., Sack M., Holland T. (2016). Putting the spotlight back on plant suspension cultures. Front. Plant Sci..

[46] Magy B., Tollet J., Laterre R., Boutry M., Navarre C. (2014). Accumulation of secreted antibodies in plant cell cultures varies according to the isotype, host species and culture conditions. Plant Biotechnol. J..

[47] De Muynck B., Navarre C., Nizet Y., Stadlmann J., Boutry M. (2009). Different subcellular localization and glycosylation for a functional antibody expressed in *Nicotiana tabacum* plants and suspension cells. Transgenic Res..

[48] Navarre C., Smargiasso N., Duvivier L., Nader J., Far J., De Pauw E., Boutry M. (2017). N-Glycosylation of an IgG antibody secreted by *Nicotiana tabacum* BY-2 cells can be modulated through co-expression of human β-1, 4-galactosyltransferase. Transgenic Res..

[49] Roulin A., Auer P.L., Libault M., Schlueter J., Farmer A., May G., Stacey G., Doerge R.W., Jackson S.A. (2013). The fate of duplicated genes in a polyploid plant genome. Plant J..

[50] Levy A.A., Feldman M. (2004). Genetic and epigenetic reprogramming of the wheat genome upon allopolyploidization. Biol. J. Linn. Soc..

[51] Gerbeau P., Güçlü J., Ripoche P., Maurel C. (1999). Aquaporin Nt-TIPa can account for the high permeability of tobacco cell vacuolar membrane to small neutral solutes. Plant J..

[52] Bots M., Vergeldt F., Wolters-Arts M., Weterings K., van As H., Mariani C. (2005). Aquaporins of the PIP2 class are required for efficient anther dehiscence in tobacco. Plant Physiol..

[53] Flexas J., Ribas-Carbó M., Hanson D.T., Bota J., Otto B., Cifre J., McDowell N., Medrano H., Kaldenhoff R. (2006). Tobacco aquaporin NtAQP1 is involved in mesophyll conductance to CO_2_ in vivo. Plant J..

[54] Otto B., Uehlein N., Sdorra S., Fischer M., Ayaz M., Belastegui-Macadam X., Heckwolf M., Lachnit M., Pede N., Priem N. (2010). Aquaporin tetramer composition modifies the function of tobacco aquaporins. J. Biol. Chem..

[55] Siefritz F., Biela A., Eckert M., Otto B., Uehlein N., Kaldenhoff R. (2001). The tobacco plasma membrane aquaporin NtAQP1. J. Exp. Bot..

[56] Azad A.K., Yoshikawa N., Ishikawa T., Sawa Y., Shibata H. (2012). Substitution of a single amino acid residue in the aromatic/arginine selectivity filter alters the transport profiles of tonoplast aquaporin homologs. Biochim. Biophys. Acta -Biomembr..

[57] Beitz E., Wu B., Holm L.M., Schultz J.E., Zeuthen T. (2006). Point mutations in the aromatic/arginine region in aquaporin 1 allow passage of urea, glycerol, ammonia, and protons. Proc. Natl. Acad. Sci. USA.

[58] Soto G., Alleva K., Mazzella M.A., Amodeo G., Muschietti J.P. (2008). AtTIP1;3 and AtTIP5;1, the only highly expressed Arabidopsis pollen-specific aquaporins, transport water and urea. FEBS Lett..

[59] Berny M.C., Gilis D., Rooman M., Chaumont F. (2016). Single mutations in the transmembrane domains of maize plasma membrane aquaporins affect the activity of monomers within a heterotetramer. Mol. Plant.

[60] Maurel C., Verdoucq L., Rodrigues O. (2016). Aquaporins and plant transpiration. Plant Cell Environ..

[61] Takano J., Wada M., Ludewig U., Schaaf G., Von Wirén N., Fujiwara T. (2006). The Arabidopsis major intrinsic protein NIP5; 1 is essential for efficient boron uptake and plant development under boron limitation. Plant Cell Online.

[62] Ma J.F. (2010). Silicon transporters in higher plants. Adv. Exp. Med. Biol..

[63] Moshelion M., Hachez C., Ye Q., Cavez D., Bajji M., Jung R., Chaumont F. (2009). Membrane water permeability and aquaporin expression increase during growth of maize suspension cultured cells. Plant Cell Environ..

[64] Zelazny E., Borst J.W., Muylaert M., Batoko H., Hemminga M.A., Chaumont F. (2007). FRET imaging in living maize cells reveals that plasma membrane aquaporins interact to regulate their subcellular localization. Proc. Natl. Acad. Sci. USA.

[65] Reisen D., Leborgne-Castel N., Özalp C., Chaumont F., Marty F. (2003). Expression of a cauliflower tonoplast aquaporin tagged with GFP in tobacco suspension cells correlates with an increase in cell size. Plant Mol. Biol..

[66] Pierman B., Toussaint F., Bertin A., Lévy D., Smargiasso N., De Pauw E., Boutry M. (2017). Activity of the purified plant ABC transporter NtPDR1 is stimulated by diterpenes and sesquiterpenes involved in constitutive and induced defenses. J. Biol. Chem..

[67] Kumar S., Stecher G., Tamura K. (2016). MEGA7: Molecular evolutionary genetics analysis version 7.0 for bigger datasets. Mol. Biol. Evol..

[68] Li B., Dewey C.N. (2011). RSEM: Accurate transcript quantification from RNA-Seq data with or without a reference genome. BMC Bioinform..

