# Supplementary material for: Evolutionary and Predictive Functional Insights into the Aquaporin Gene Family in the Allotetraploid Plant Nicotiana tabacum
Source: Int J Mol Sci. 2020 Jul 3;21(13):4743. doi: 10.3390/ijms21134743 (PMC7370101; doi:10.3390/ijms21134743)
Supplement: Supplementary file 1 [file ijms-21-04743-s001.pdf]

Figure S1. Multiple sequence alignment of NtPIPs. The amino acid sequences were aligned using the Clustal Omega server. The two conserved NPA motifs are shown in yellow, the residues at H2, H5, LE1, and LE2 of the ar/R filter are in green, FPs (P1-P5) are in cyan, TM are shaded with grey background.

```
NtPIP1;3       ----MAENKEEDVKLGANKYRETQPLGTAAQT-DKDYKEPPPAPLVWRQKSCRHGLF-TE  54
NtPIP1;1       ----MAENKEEDVNLGANKYRETQPLGTAAQTENKDYIEPPPAPLFEPGELSSWSFYRAG  56
NtPIP1;6       ----MAENKEEDVNLGANKYRETQPLGTAAQTENKDYIEPPPAPLFEPGELSSWSFYRAG  56
NtPIP1;2       ----MAENKEEDVKLGANKFRETQPLGTAAQT-DKDYKEPPPAPLFEPGELSSWSFYRAG  55
NtPIP1;4       ----MAENKEEDVKLGANKFRETQPLGTAAQT-DKDYKEPPPAPLFEPGELSSWSFYRAG  55
NtPIP1;5       ----MAENKEEDVKLGANKFRETQPLGTAAQT-DKDYKEPPPAPLFEPGELSSWSFYRAG  55
NtPIP1;7       ----MAENKEEDVKLGANKYRETQPLGTAAQT-DKDYKEPPPAPLFEPGELSSWSFYRAG  55
NtPIP1;8       ----MAENKEEDVKLGANKYRETQPLGTAAQT-DKDYKEPPPAPLFEPGELSSWSFYRAG  55
NtPIP1;11      -----MEGKEEDVKVGANKYSERQPLGTSS--QSKDYKEPPPAPLFEPGELHSWSFWRAG  53
NtPIP1;12      -----MEGKEEDVKVGANKYSERQPLGTSA--QSKDYKEPPPAPLFEPGELHSWSFWRAG  53
NtPIP1;13      -----MEGKEEDVKVGANKYSERQPLGTSA--QSKDYKEPPAAPLFEPGELHSWSFWRAG  53
NtPIP1;9       -----MENKEEDVRLGANKYSERQAIGTAAQ-SDKDYKEPPPAPLFEAGELTSWSFYRAG  54
NtPIP1;10      -----MEHREEDVRVGANKYSERQAIGTAAHSQDKDYKEPPPAPLFEPGELMSWSFYRAG  55
NtPIP2;9       --------MAKNV-------------NSEGSFTTKDYQDPPPAPLIDPEELTQWSFYRAL  39
NtPIP2;10      --------MAKNV-------------GSEGSFTTKDYQDPPPAPLIDPEELTQWSFYRAL  39
NtPIP2;17      --------MSKEVEAV---------SEQPAEYSAKDYTDPPPTPLIDFEELTKWSLYRAC  43
NtPIP2;18      --------MTKEVEAV---------SEQPAEYSAKDYTDPPPTPLIDFEELTKWSLYRAC  43
NtPIP2;19      ------------MGKD---------VEAATEFSAKDYTDPPPAPLIDFEELKQWSFYRAA  39
NtPIP2;20      --------MTKEVEVA---------TEQPTEFSAKDYTDPPPAPLVDFEELTQWSLYRAV  43
NtPIP2;21      --------MTKEVEVA---------REQAAEFSAKDYTDPPPAPLVDFEELTQWSLYRAV  43
NtPIP2;11      --------MAKDIEYG---------TDQ--YAPNKDYQDPPPAPLIDAEELGKWSFYRAI  41
NtPIP2;12      --------MAKDIEYG---------TDQ--YAPNKDYQDPPPAPLIDAEELGKWSFYRAI  41
NtPIP2;13      MSLFFGEQMAKDTEVG---------TE----YAPKDYQDPPPAPLIDPEELGKWSFYRAI  47
NtPIP2;15      --------MGKDIEVG---------TE----YAPKDYQDPPPAPLIDPEELGKWSFYRAI  39
NtPIP2;14      MSLFFGEQMAKDTEVG---------TE----YAPKDYQDPPPAPLIDPEELGKWSFYRAI  47
NtPIP2;16      --------MGKDIEVG---------TE----YAPKDYQDPPPAPLIDPEELGKWSFYRAI  39
NtPIP2;3       --------MSKDV-IE---------EGQVHQQHGKDYVDPPPAPLLDFAELKLWSFYRAL  42
NtPIP2;1       --------MSKDV-IE---------EGQVHQQHGKDYVDPPPAPLLDFAELKLWSFHRAL  42
NtPIP2;2       --------MSKDV-IE---------EGQVHQQHGKDYVDPPPAPLLDFAELKLWSFHRAL  42
NtPIP2;4       --------MSKDV-IE---------EGQTH-QHGKDYVDPPPAPLLDMAELTKWSFYRAV  41
NtPIP2;5       --------MSKDV-IE---------EGQTH-QHGKDYVDPPPAPLLDMAELTKWSFYRAV  41
NtPIP2;7       --------------------------------MVKDYVDKPAAPLFDTVEVKKWSFYRAL  28
NtPIP2;6       --------------------------------MTRDYVDPPAAPLFDMAELKKWSFYRAL  28
NtPIP2;8       --------------------------------MARDYVDPPAAPLFDTAELKKWSFYRAL  28
                                                  :** : * :**.   :    .:  :

                    TM1                                      H2  TM2
NtPIP1;3       LEFSMATFLFLYITILTVMGLKRS------DSLCSSVGIQGVAWAFGGMIFALVYCTAGI  108
NtPIP1;1       IAEFMATFLFLYITILTVMGLKRS------DSLCSSVGIQGVAWAFGGMIFALVYCTAGI  110
NtPIP1;6       IAEFMATFLFLYITILTVMGLKRS------DSLCSSVGIQGVAWAFGGMIFALVYCTAGI  110
NtPIP1;2       IAEFMATFLFLYITILTVMGLKRS------DSLCSSVGIQGVAWAFGGMIFALVYCTAGI  109
NtPIP1;4       IAEFMATFLFLYITILTVMGLKRS------DSLCSSVGIQGVAWAFGGMIFALVYCTAGI  109
```

```
NtPIP1;5      IAEFMATFLFLYITILTVMGLKRS------DSLCSSVGIQGVAWAFGGMIFALVYCTAGI 109
NtPIP1;7      IAEFMATFLFLYITILTVMGLKRS------DSLCSSVGIQGVAWAFGGMIFALVYCTAGI 109
NtPIP1;8      IAEFMATFLFLYITILTVMGLKRS------DSLCSSVGIQGVAWAFGGMIFALVYCTAGI 109
NtPIP1;11     IGEFMPTFLFLYITVLTVMGYSRA------NSKCSTVGVQGIVWAFGGMIFALVYCTAGI 107
NtPIP1;12     IAEFMATFLFLYITVLTVMGYSRA------NSKCSTVGVQGIAWAFGGMIFALVYCTAGI 107
NtPIP1;13     IAEFMATFLFLYITVLTVMGYSRA------NSKCSTVGVQGIAWAFGGMIFALVYCTAGI 107
NtPIP1;9      IAEFMATFLFLYITILTVMGVSKS------ESKCSTVGIQGIAWAFGGMIFALVYCTAGI 108
NtPIP1;10     IAEFMATFLFLYITVLTVMGVSKS------ESKCPTVGIQGIAWAFGGMIFALVYCTAGI 109
NtPIP2;9      IAEFIATLLFLYITVLTVIGYKNQSAT--TSDPCAGVGVLGIAWAFGGMIFVLVYCTAGI 97
NtPIP2;10     IAEFIATLLFLYITVLTVIGYKSQSAT--ATDPCAGVGILGIAWAFGGMIFVLVYCTAGI 97
NtPIP2;17     IAEFIATLLFLYVTVLTVIGYKHQSDTKDGGDICGGVGILGIAWAFGGMIFVLVYCTAGI 103
NtPIP2;18     IAEFIATLLFLYVTVLTVIGYKHQSDTKDGGDICGGVGILGIAWAFGGMIFVLVYCTAGI 103
NtPIP2;19     IAEFIATLLFLYVTVLTVIGYKHQSDVDANGDVCGGVGILGIAWAFGGMIFVLVYCTAGI 99
NtPIP2;20     IAEFIATLLFLYVTVLTVIGYKVQSDVKADGDICGGVGILGIAWAFGGMIFILVYCTAGI 103
NtPIP2;21     IAEFIATLLFLYVTVLTVIGYKVQSDVKADGDICGGVGILGIAWAFGGMIFILVYCTAGI 103
NtPIP2;11     IAEFIATLLFLYITVLTVIGYKSQSDTKHNGDECGGVGILGIAWAFGGMIFVLVYCTAGI 101
NtPIP2;12     VAEFIATLLFLYITVLTVIGYKSQSDTKHNGDECGGVGILGIAWAFGGMIFVLVYCTAGI 101
NtPIP2;13     IAEFIATLLFLYITVLTVIGYKSQIDPDHNGEQCGGVGILGIAWAFGGMIFVLVYCTAGI 107
NtPIP2;15     IAEFIATLLFLYITVLTVIGYKSQIDPDHNGEQCGGVGILGIAWAFGGMIFVLVYCTAGI 99
NtPIP2;14     IAEFIATLLFLYITVLTVIGYKSQTDTDHNGEQCGGVGILGIAWAFGGMIFVLVYCTAGI 107
NtPIP2;16     IAEFIATLLFLYITVLTVIGYKSQTDTDHNGEQCGGVGILGIAWAFGGMIFVLVYCTAGI 99
NtPIP2;3      IAEFIATLLFLYVTVATVIGHKKLN----GADKCDGVGILGISWAFGGMIFVLVYCTAGI 98
NtPIP2;1      IAEFIATLLFLYVTVATVIGHKKLN----GADKCDGVGILGISWAFGGMIFVLVYCTAGI 98
NtPIP2;2      IAEFIATLLFLYVTVATVIGHKKLN----GADKCDGVGILGISWAFGGMIFVLVYCTAGI 98
NtPIP2;4      IAEFIATLLFLYVTVATVIGHKKLN----AADHCDGVGILGIAWAFGGMIFVLVYCTAGI 97
NtPIP2;5      IAEFIATLLFLYVTVATVIGHKKLN----AADHCDGVGILGIAWAFGGMIFVLVYCTAGI 97
NtPIP2;7      IAEFVATLLFLYVSVATVIGHKKQV----G--PCDGVGLLGIAWAFGGMIFVLVYCTAGI 82
NtPIP2;6      IAEFVATFLFLYVSVATVIGHKKQV----G--PCDGVGLLGIAWAFGGMIFVLVYCTAGI 82
NtPIP2;8      IAEFVATFLFLYVSVATVIGHKKQV----G--PCDGVGLLGISWAFGGMIFVLVYCTAGI 82
              :   : *:****::: **:* .          *  **: *: ******** ********
                                       TM3                P1
NtPIP1;3      SGGHINPAVTFGLFLARKLSLTRAVFYMVMQCLGAICGAGVVKGFMVGPYQRLGGGANVV 168
NtPIP1;1      SGGHINPAVTFGLFLARKLSLTRAVFYMVMQCLGAICGAGVVKGFMKGPYQRLGGGANVV 170
NtPIP1;6      SGGHINPAVTFGLFLARKLSLTRAVFYMVMQCLGAICGAGVVKGFMKGPYQRLGGGANVV 170
NtPIP1;2      SGGHINPAVTFGLFLARKLSLTRAVFYIVMQCLGAICGAGVVKGFMVGPYQRHGGGANVV 169
NtPIP1;4      SGGHINPAVTFGLFLARKLSLTRAIFYIVMQCLGAICGAGVVKGFMVGPYQRLGGGANVV 169
NtPIP1;5      SGGHINPAVTFGLFLARKLSLTRAIFYIVMQCLGAICGAGVVKGFMVGPYQRLGGGANVV 169
NtPIP1;7      SGGHINPAVTFGLFLARKLSLTRALFYMVMQCLGAICGAGVVKGFMVGPYQRLGGGANMV 169
NtPIP1;8      SGGHINPAVTFGLFLARKLSLTRALFYMVMQCLGAICGAGVVKGFMVGPYQRLGGGANVV 169
NtPIP1;11     SGGHINPAVTFGLFLARKLSLTRAVFYIVMQCLGAICGAGVVKGFQPSLYQVKGGGANVV 167
NtPIP1;12     SGGHINPAVTFGLFLARKLSLTRAVFYIVMQCLGAICGAGVVKGFQPSLYQVKGGGANVV 167
NtPIP1;13     SGGHINPAVTFGLFLARKLSLTRAVFYIVMQCLGAICGAGVVKGFQPSLYQVKGGGANIM 167
NtPIP1;9      SGGHINPAVTFGLFLARKLSLTRAVFYMVMQCLGAICGAGVVKGFGKTLYQTKGGGANVV 168
NtPIP1;10     SGGHINPAVTFGLFLARKLSVTRALFYMVMQCLGAICGAGVVKGFGKTLYQTKGGGANVV 169
NtPIP2;9      SGGHINPAVTFGLFLARKVSLIRALMYMVAQCLGAICGVGLVKAFQSAYYHRYDGGANML 157
NtPIP2;10     SGGHINPAVTFGLFLARKVSLIRAVMYMVAQCLGAICGVGLVKAFQSAYYHRYDGGANML 157
```

```
NtPIP2;17      SGGHINPAVTFGLFLARKVSLIRAVLYMVSQCLGAICGVGLVKAFQKAYFNRYGGGVNVM 163
NtPIP2;18      SGGHINPAVTFGLFLARKVSLMRAVLYMVSQCLGAICGVGLVKAFQKAYYNRYGGGVNVM 163
NtPIP2;19      SGGHINPAVTFGLFLARKVSLIRALVYMVAQCLGAICGVGFVKAFQSAYYDRYGGGANVM 159
NtPIP2;20      SGGHINPAVTFGLFLARKVSLIRAVLYMVAQCLGAICGVGFVKAFQSAYYVRYGGGANVM 163
NtPIP2;21      SGGHINPAVTFGLFLARKVSLIRAVLYMVAQCLGAICGVGLVKGFQSAYYVRYGGGANVM 163
NtPIP2;11      SGGHINPAVTFGLFLARKISLARAVMYMIAQCLGAICGCGLVKAFQKSYYVRYGGGANEL 161
NtPIP2;12      SGGHINPAVTFGLFLARKISLARAVMYMIAQCLGAICGCGLVKAFQKSYYVNYGGGANEL 161
NtPIP2;13      SGGHINPAVTFGLFLARKVSLVRAIMYMLAQCLGAICGCGLVKAFQKAYYVKYGGGANTL 167
NtPIP2;15      SGGHINPAVTFGLFLARKVSLVRAIMYMLAQCLGAICGCGLVKAFQKAYYVKYGGGANTL 159
NtPIP2;14      SGGHINPAVTFGLFLARKVSLVRAIMYMLAQCLGAICGCGLVKAFQKAYYVKYGGGANML 167
NtPIP2;16      SGGHINPAVTFGLFLARKVSLVRAIMYMLAQCLGAICGCGLVKAFQKAYYVKYGGGANML 159
NtPIP2;3       SGGHINPAVTFGLFLARKVSLLRAVGYIIAQSLGAICGVGLVKGFMKHYYNTLGGGANFV 158
NtPIP2;1       SGGHINPAVTFGLFLARKVSLLRAVGYIIAQSLGAICGVGLVKGFMKHYYNTLGGGANFV 158
NtPIP2;2       SGGHINPAVTFGLFLARKVSLLRAVGYIIAQSLGAICGVGLVKGFMKHYYNTLGGGANFV 158
NtPIP2;4       SGGHINPAVTFGLLLARKVSLIRAVAYIIAQSLGAICGVGFVKAFMKHYYNLEGGGANFV 157
NtPIP2;5       SGGHINPAVTLGLFLARKVSLIRAVAYIIAQSLGAICGVGFVKAFMKHYYNLEGGGANFV 157
NtPIP2;7       SGGHINPAVTFGLLLARKVSLLRAVGYMVAQCLGAICGVGLVKGFMKHDYNTYGGGANTV 142
NtPIP2;6       SGGHINPAVTFGLLLARKVSLLRAVGYMVAQCLGAICGVGLVKGFMKHDYNTYGGGANTV 142
NtPIP2;8       SGGHINPAVTFGLLLARKVSLLRAVAYMVAQCLGAICGVGLVKGFMKHDYNTHGGGANTV 142
               **********:**:****:*: **: *:: *.****** *.**.*     :    .**.* :
                               TM4                                   TM5  H5
NtPIP1;3       NPGYTKGDGLGAEIIGTFVLVYTVFSATDAKRNARDSHVPILAPLPIGFAVFLVHLATIP 228
NtPIP1;1       NPGYTKGDGLGAEIIGTFVLVYTVFSATDAKRNARDSHVPILAPLPIGFAVFLVHLATIP 230
NtPIP1;6       NPGYTKGDGLGAEIIGTFVLVYTVFSATDAKRNARDSHVPILAPLPIGFAVFLVHLATIP 230
NtPIP1;2       NSGYTKGDGLGAEIIGTFVLVYTVFSATDAKRNARDSHVPILAPLPIGFAVFLVHLATIP 229
NtPIP1;4       NHGYTKGDGLGAEIIGTFVLVYTVFSATDAKRNARDSHVPILAPLPIGFAVFLVHLATIP 229
NtPIP1;5       NHGYTKGDGLGAEIIGTFVLVYTVFSATDAKRNARDSYVPILAPLPIGFAVFLVHLATIP 229
NtPIP1;7       QPGYTKGDGLGAEIIGTFVLVYTVFSATDAKRNARDSHVPILAPLPIGFAVFLVHLATIP 229
NtPIP1;8       QPGYTKGDGLGAEIVGTFVLVYTVFSATDAKRNARDSHVPILAPLPIGFAVFLVHLATIP 229
NtPIP1;11      NNDYTKGDGLGAEIVGTFVLVYTVFSATDAKRNARDSHVPILAPLPIGFAVFLVHLATIP 227
NtPIP1;12      NHGYTKGDGLGAEIVGTFVLVYTVFSATDAKRNARDSHVPILAPLPIGFAVFLVHLATIP 227
NtPIP1;13      NHGYTKGDGLGAEIVGTFVLVYTVFSATDAKRNARDSHVPILAPLPIGFAVFLVHLATIP 227
NtPIP1;9       NLGYTKGSGLGAEIVGTFVLVYTVFSATDAKRSARDSHVPILAPLPIGFAVFLVHLATIP 228
NtPIP1;10      NPGYTKGDGLGAEIVGTFVLVYTVFSATDAKRSARDSHVPILAPLPIGFAVFLVHLATIP 229
NtPIP2;9       SDGYSHGVGLSAEIIGTFVLVYTVFSATDPKRNARDSHVPVLAPLPIGFAVFMVHLATIP 217
NtPIP2;10      SDDYSHGVGLSAEIIGTFVLVYTVFSATDPKRNARDSHVPVLAPLPIGFAVFMVHLATIP 217
NtPIP2;17      AGGHNKGVGLGAEIIGTFVLVYTVFSATDPKRSARDSHVPVLAPLPIGFAVFMVHLATIP 223
NtPIP2;18      AGGHNKGVGLGAEIIGTFVLVYTVFSATDPKRSARDSHVPVLAPLPIGFAVFMVHLATIP 223
NtPIP2;19      AAGHTKGVGLAAEIIGTFVLVYTVFSATDPKRSARDSHVPVLAPLPIGFAVFMVHLATIP 219
NtPIP2;20      APGHTKGVGLAAEIIGTFVLVYTVFSATDPKRNARDSHVPVLAPLPIGFAVFMVHLATIP 223
NtPIP2;21      APGHTKGVGLAAEIIGTFVLVYTVFSATDPKRNARDSHVPVLAPLPIGFAVFMVHLATIP 223
NtPIP2;11      ATGYSTGTGLAAEIIGTFVLVYTVFSATDPKRNARDSHVPVLAPLPIGFAVFMVHLATIP 221
NtPIP2;12      ATGYSTGTGLAAEIIGTFVLVYTVFSATDPKRNARDSHVPVLAPLPIGFAVFMVHLATIP 221
NtPIP2;13      NDGYNTGTGLGAEIIGTFVLVYTVFAATDPKRNARDSHVPVLAPLPIGFAVFMVHLATIP 227
NtPIP2;15      NDGYSTGTGLGAEIIGTFVLVYTVFAATDPKRNARDSHVPVLAPLPIGFAVFMVHLATIP 219
NtPIP2;14      NDGYSTGTGLGAEIIGTFVLVYTVFAATDPKRNARDSHVPVLAPLPIGFAVFMVHLATIP 227
```

```
NtPIP2;16       NDGYSTGTGLGAEIIGTFVLVYTVFAATDPKRNARDSHIPVLAPLPIGFAVFMVHLATIP 219
NtPIP2;3        QPGYNKGTALGAEIIGTFVLVYTVFSATDPKRSARDSHVPVLAPLPIGFAVFMVHLATIP 218
NtPIP2;1        QPGYNKGTALGAEIIGTFVLVYTVFSATDPKRSARDSHVPVLAPLPIGFAVFMVHLATIP 218
NtPIP2;2        QPGYNKGTALGAEIIGTFVLVYTVFSATDPKRSARDSHVPVLAPLPIGFAVFMVHLATIP 218
NtPIP2;4        QPGYNKGTALGAEIIGTFVLVYTVFSATDPKRSARDSHVPVLAPLPIGFAVFMVHLATIP 217
NtPIP2;5        QPGYNKGTALGAEIIGTFVLVYTVFSATDPKRSARDSHVPVLAPLPIGFAVFMVHLATIP 217
NtPIP2;7        AVGYSTGTALGAEIIGTFVLVYTVFSATDAKSKARDSHVPVLAPLPIGFSVFMVHLATIP 202
NtPIP2;6        AVGYSTGTALGAEIIGTFVLVYTVFSATDPKRNSRDSHVPVLAPLPIGFAVFMVHLATIP 202
NtPIP2;8        AVGYSTGAALGAEIIGTFVLVYTVFSATDPKRNSRDSHVPVLAPLPIGFAVFMVHLATIP 202
                   .:. * .*.***:**********:*** *  .:***::*:********:**:*******
                  LE1  LE2P2 P3              P4P5  TM6
NtPIP1;3        ITGTGITPARSLGAAIIFNQDRAWDDHWIFWVGPFIGAALAAVYHQIIIRAIAFKS---- 284
NtPIP1;1        ITGTGINPARSLGAAIIFNKKQAWDDHWIFWVGPFIGAALAAVYHQIIIRAIPFKS---- 286
NtPIP1;6        ITGTGINPARSLGAAIIFNKKQAWDDHWIFWVGPFIGAALAAVYHQIIIRAIPFKSKA-- 288
NtPIP1;2        ITGTGINPARSLGAAIIYNTDQAWDDHWIFWVGPFIGAALAAVYHQIIIRAIPFHKSS-- 287
NtPIP1;4        ITGTGINPARSLGAAIIYNTDQAWDDHWIFWVGPFIGAALAAVYHQIIIRAIPFHKSS-- 287
NtPIP1;5        ITGTGINPARSLGAAIIYNTDQAWDDHWIFWVGPFIGAALAAVYHQIIIRAIPFHKSS-- 287
NtPIP1;7        ITGTGINPARSLGAAIIFNQDQAWDDHWIFWVGPFIGAALAAVYHQIIIRAIPFKSKS-- 287
NtPIP1;8        ITGTGINPARSLGAAIIFNQDRAWDDHWIFWVGPFIGAALAAVYHQIIIRAIPFKSKS-- 287
NtPIP1;11       IIGTCINPARSLGAAIIYNRDQAWDDH--------------------------------- 254
NtPIP1;12       ITGTGINPARSLGAAIIYNRDQAWDDHWIFWVGPFVGAALAALYHQVIIRAIPFKSGNLA 287
NtPIP1;13       ITGTGINPARSLGAAIIYNRDQAWDDHWIFWVGPFIGAALAALYHQVIIRAIPFRSGN-- 285
NtPIP1;9        ITGTGINPARSLGAAIIYNQDHAWDDHWIFWVGPFIGAALAALYHQVVIRAIPFKSK--- 285
NtPIP1;10       ITGTGINPARSLGAAIIYNKEHAWNDHWIFWVGPFIGAALAALYHQVVIRAIPFKSK--- 286
NtPIP2;9        ITGTGINPARSLGAAVIFNQHKAWKDHWIFWVGPFIGAAIAAFYHQFILRAGAAKALGSF 277
NtPIP2;10       ITGTGINPARSLGAAVIFNQHKAWKDHWIFWVGPFIGAAIAAFYHQFILRAGAAKALGSF 277
NtPIP2;17       ITGTGINPARSFGAAVIYNQDKAWDEHWIFWVGPFVGAFAAAVYHQYILRAGALKALGSF 283
NtPIP2;18       ITGTGINPARSFGAAVIYNQDKAWDEHWIFWVGPFVGAFAAAVYHQYILRAGALKALGSF 283
NtPIP2;19       ITGTGINPARSFGAAVIYNQDKAWDEHWIFWVGPFIGAFAAAAYHQYILRAGAVKALGSF 279
NtPIP2;20       ITGTGINPARSFGAAVIYNHDKAWDEHWIFWVGPFIGAFAAAVYHQFILRAGAIKALGSF 283
NtPIP2;21       ITGTGINPARSFGAAVIYNQDKAWDEHWIFWVGPFIGAFAAAVYHQFILRAGAIKALGSF 283
NtPIP2;11       ITGTGINPARSFGAAVIYGKDKAWDDQWIFWVGPLIGAAIAALYHQYILRAGAVKALGSF 281
NtPIP2;12       ITGTGINPARSFGAAVIYGKEKAWDDQWIFWVGPFIGAAIAALYHQYILRAGAVKALGSF 281
NtPIP2;13       VTGTGINPARSFGAAVIYGKEKAWDDQWIFWVGPFIGAAIAAFYHQFILRAGAVKALGSF 287
NtPIP2;15       VTGTGINPARSFGAAVIYGKEKAWDDQWIFWVGPFIGAAIAAFYHQFILRAGAVKALGSF 279
NtPIP2;14       VTGTGINPARSFGAAVIYGKDKAWDDQWIFWVGPFIGAAIAAFYHQFILRAGAVKALGSF 287
NtPIP2;16       VTGTGINPARSFGAAVIYGKEKAWDDQWIFWVGPFIGAAIAAFYHQFILRAGAVKALGSF 279
NtPIP2;3        ITGTGINPARSFGAAVIYNTEKVWDDQWIFWVGPFVGALVAAVYHQYILRGSAIKALGSF 278
NtPIP2;1        ITGTGINPARTFGAAVIYNTEKIWDDQWIFWVGPFVGALVAAVYHQYILRGSAIKALGSF 278
NtPIP2;2        ITGTGINPARSFGAAVIYNTEKIWDDQWIFWVGPFVGALVAAVYHQYILRGSAIKALGSF 278
NtPIP2;4        ITGTGINPARSFGAAVIYNKEKIWDDQWIFWVGPFVGALIAAIYYQFVLRAGAVKALGSF 277
NtPIP2;5        ITGTGINPARSFGAAVIYNKEKIWDDQWIFWVGPFVGALIAAIYHQFVLRAGAVKALGSF 277
NtPIP2;7        ITGTGINPARSFGAAVIYNNSTAWNDHWIFWVGPFVGALVASLYHQQVLRAHVLDTWNSY 262
NtPIP2;6        ITGTGINPARSFGAAVIYNDTTAWNDHWIFWVGPSLGALAAALYYQQVLRAQAAKTLSSF 262
NtPIP2;8        ITGTGINPARSFGAAVIYNHTTAWNDHWIFWVGPFLGALAAALYYQQLLRAQAAKTLSSF 262
                : ** *.***::***:*:.     *.::
```

NtPIP1;3	------	284
NtPIP1;1	------	286
NtPIP1;6	------	288
NtPIP1;2	------	287
NtPIP1;4	------	287
NtPIP1;5	------	287
NtPIP1;7	------	287
NtPIP1;8	------	287
NtPIP1;11	------	254
NtPIP1;12	------	287
NtPIP1;13	------	285
NtPIP1;9	------	285
NtPIP1;10	------	286
NtPIP2;9	RSSSQV	283
NtPIP2;10	RSSSQV	283
NtPIP2;17	RSNA--	287
NtPIP2;18	RSNA--	287
NtPIP2;19	RSNA--	283
NtPIP2;20	RSNA--	287
NtPIP2;21	RSNA--	287
NtPIP2;11	RSNA--	285
NtPIP2;12	RSNA--	285
NtPIP2;13	RSNA--	291
NtPIP2;15	RSNA--	283
NtPIP2;14	RSNA--	291
NtPIP2;16	RSNA--	283
NtPIP2;3	RSNPTN	284
NtPIP2;1	RSNPTN	284
NtPIP2;2	RSNPTN	284
NtPIP2;4	RSNPTN	283
NtPIP2;5	RSNPTN	283
NtPIP2;7	YNNPST	268
NtPIP2;6	HSNPSI	268
NtPIP2;8	HSNSSI	268

Figure S2. Multiple sequence alignment of NtTIPs. The amino acid sequences were aligned using the Clustal Omega server. The two conserved NPA motifs are shown in yellow, the residues at H2, H5, LE1, and LE2 of the ar/R filter are in green, FPs (P1-P5) are in cyan, TM are shaded with grey background.

```
                                          TM1
NtTIP5;1        -----MASLASRLQHSVTPNALRSYVAEFLSTFLFVFAAAGAAMSTRKMTPDATSDPSSL  55
NtTIP5;2        -----MASLASRLQHSVTPNALRSYVAEFLSTFIFVFAAAGAAMSTRKMTPDATSDPSSL  55
NtTIP4;1        ----MAKIAVGSSREAIQPDCIQALIVEFICTFLFVFAGVGSAMAANKLNGDPLV---SL  53
NtTIP4;2        ----MAKIAVGNSREAIQPDCIQALIVEFIVTFLFVFAGVGSAMAANKLNGDPLV---SL  53
NtTIP3;1        MAMPERRYAFGRADEATHPDSMRATLSELLSTFIFVFAGEGTVLALDKLYPETALGASRL  60
NtTIP3;2        MAMPARRYAFGRADEATHPDSMRATLSELLSTFIFVFAGEGTVLALDKLYPETALGASRL  60
NtTIP3;3        -MQPPRRYAFGRVDEATHPDSMRATLSEFLSTFIFVFAGEGSALALDKLYPDTALGASRL  59
NtTIP3;4        -MQPPRRYAFGRVDEATHPDSMRATLSEFLSTFIFVFAGEGSALALDKLYPDTALGASRL  59
NtTIP2;4        ----MPCIAFGRFDDSVSSGSIKAYVAEFISTLLFVFAGVGSAIAYNKLTADAALDPAGL  56
NtTIP2;5        ----MPCIALGRFDDSFSSGSIKAYIAEFISTLLFVFAGVGSAIAYNKLTADAALDPAGL  56
NtTIP2;3        ----MPAIAFGRFDDSFSLGSIKAYIAEFISTLLFVFAGVGSAIAYNKLTADAALDPSGL  56
NtTIP2;1        ----MPGIAFGRIDDSFSVGSLKAYLAEFISTLLFVFAGVGSAIAYNKLTADAALDPAGL  56
NtTIP2;2        ----MPCIAFGRIDDSFSVGSLKAYLAEFISTLLFVFAGVGSAIAYNKLTADAALDPAGL  56
NtTIP2;8        ----MVRIAFGSIGDSFSVGSLKAYVAEFIATLLFVFAGVGSAIAYNKLTADAALDPAGL  56
NtTIP2;9        ----MVKIAFGSIGDSFSVGSLKAYVAEFIATLLFVFAGVGSAIAYNKLTADAALDPAGL  56
NtTIP2;10       ----MVLIAFGSIGDSFSVGSLKAYVAEFIATLLFVFAGVGSAIAYNKLTADAALDPAGL  56
NtTIP2;6        ----MVKIAFGNYNDSVSAASIKAYLAEFIATLLFVFAGVGSVISYNKLTTDAALDPAGL  56
NtTIP2;7        ----MAKIAFGNYNDSVSAASIKAYLAEFIATLLFVFAGVGSAISYNKLTTDAALDPAGL  56
NtTIP1;4        --MPIHQITIGTHEELRQPGALKAALAEFISTLIFVFAGQGSGMAFNKLTSDSTNTPAGL  58
NtTIP1;3        --MPIHQIAVGSHEELRQSGTLKAALAEFICTLIFVFAGQGSGMAFNKLSADGTATPAGL  58
NtTIP1;1        --MPINQIAVGSHEELRQPGTLKAALAEFICTLIFVFAGQGSGMAFNKLSVDGTATPSGL  58
NtTIP1;2        --MPIHQIAVGSHEELRQPGTLKAALAEFICTLIFVFAGQGSGMAFNKLSVDGTATPSGL  58
NtTIP1;5        --MPISKISLGNLAEASQPDALKAALAEFISMLIFVFAGEGSGMAFGKLTNGGAATPAGL  58
NtTIP1;6        --MPISKITLGNLAEASQPDALKAALAEFISMLIFVFAGEGSGMAFGKLTNGGAATPAGL  58
NtTIP1;7        --MPFSRIAVGRPEEATHSDALKAALAEFISTLIFVFAGSGSGVAFSKLTDGGANTPAGL  58
NtTIP1;8        --MPISRIAIGRPEEATHPDALKAALAEFISTLIFVFAGSGSGVAFSKLTGGGANTPAGL  58
NtTIP1;9        --MPISRIAIGRPEEATHPDALKAALAEFISTLIFVFAGSGSGVAFSKLTGGGANTPAGL  58
                          .   .      ::: : *::  ::****. *: ::  *:          *

                 TM2  H2                           TM3
NtTIP5;1        VAVAVANAFALSVAVYISANISGGHVNPAVTFGMAIGGHISIPMSIFYWISQMLGSVMAC  115
NtTIP5;2        VAVAVANAFALSVAVYISANISGGHVNPAVTFGMAIGGHISIPMSIFYWISQMLGSVMAC  115
NtTIP4;1        FFVAMAHALVVAVTISAGFRISGGHLNPAVTLGLCMGGHITVFRSILYWIDQLLASVAAC  113
NtTIP4;2        FFVAMAHALVVAVTISAGFRISGGHLNPAVTLGLCMGGHITVFRSILYWIDQLLASVAAC  113
NtTIP3;1        TAIALAHALSFFAAVASSLNVSGGHINPAVTFGALVGGRISVVRAVYYWLAQLVGAVVAS  120
NtTIP3;2        TAIALAHALSFFAAVASSLNVSGGHINPAVTFGALVGGRISVVRAVYYWLAQLVGAVIAS  120
NtTIP3;3        TAIALAHALSLFAAVASSMNVSGGHINPAVTFGALVGGRISVLRAVYYWVAQLLGAVVAS  119
NtTIP3;4        TAIALAHALSLFAAVASSMNVSGGHINPAVTFGALVGGRVSVLRAVYYWVAQLLGAVVAS  119
NtTIP2;4        VAVAVCHGLALFVAVAIAANISGGHVNPAVTFGLALGGQITIITGLFYWIAQLLGAIAAS  116
NtTIP2;5        VAVAVCHGLALFVAVAIAANISGGHVNPAVTFGLALGGQITIITGLFYWIAQVLGAIAAS  116
NtTIP2;3        VAVAVCHGFALFVAVSVGANISGGHVNPAVTFGLALGGQITLLTGLFYWIAQLLGATVAS  116
NtTIP2;1        VAVAVCHGFGLFVAVAVGANISGGHVNPAVTFGLALGGQITILTGLFYIIAQLLGSIVAC  116
```

```
NtTIP2;2     VAVAVCHGFALFVAVAIGANISGGHVNPAVTFGLALGGQITLLTGLFYTIAQLLGSIVAC 116
NtTIP2;8     VAVAVAHAFALFVGVSIAANISGGHLNPAVTLGLAVGGNITILTGFFYWIAQLLGSTVAC 116
NtTIP2;9     VAVAVAHAFALFVGVSIAANISGGHLNPAVTLGLAVGGNITILTGFFYWIAQLLGSTVAC 116
NtTIP2;10    VAVAVAHAFALFVGVSIAANISGGHLNPAVTLGLAVGGNITILTGFFYWIAQLLGSTVAC 116
NtTIP2;6     VAVAVAHAFALFVGVSMAANISGGHLNPAVTFGLAVGGNITILTGFFYWIAQLLGSTIAC 116
NtTIP2;7     VAVAVAHAFALFVGVSMAANISGGHLNPAVTFGLAVGGNITILTGFFYWIAQLLGSTVAC 116
NtTIP1;4     IAAAVAHAFGLFVAVSVSFNISGGHVNPAVTFGAFIGGNITFFRGILYIIAQLLGSTVAC 118
NtTIP1;3     ISASIAHAFGLFVAVSVGANISGGHVNPAVTFGAFVGGNITLFRGILYIVAQLLGSTVAC 118
NtTIP1;1     ISASIAHAFGLFVAVSVGANISGGHVNPAVTFGAFVGGNITLFRGILYIIAQLLGSTVAC 118
NtTIP1;2     ISASIAHAFGLFVAVSVGANISGGHVNPAVTFGAFVGGNITLFRGILYITAQLLGSTVAC 118
NtTIP1;5     ISAAIAHAFALFVAVSVGANISGGHVNPAVTFGAFVGGHITLFRSVLYWIAQLLGSVVAC 118
NtTIP1;6     ISAAIAHAFALFVAVSVGANISGGHVNPAVTFGAFVGGHITLFRSVLYWIAQLLGSVVAC 118
NtTIP1;7     IAAAIAHAFALFVVVSVGANISGGHVNPAVTFGAFVGGNISLLRGILYWIAQLLGSVVAC 118
NtTIP1;8     IAAAIAHAFGLFVAVSVGANISGGHVNPAVTFGAFVGGNITLLRGILYWIAQLLGSVVAC 118
NtTIP1;9     IAAAIAHAFGLFVAVSVGANISGGHVNPAVTFGAFVGGNITLLRGILYWIAQLLGSVVAC 118
               ::.:.: . . :  . .:****:*****:*  :**.::.  .. *   *::.:  *.

                                                TM4
NtTIP5;1     LVLKCTNQQV--QTHGIPHDMTGFGGAVLEGVMTFGLVYTVYAA-ADPRRCVHGAIGPLT 172
NtTIP5;2     LVLKCTNQQV--QTHGIPHDMTGFGGAVLEGVMTFGLVYTVYAA-ADPRRCVHGAIGPLA 172
NtTIP4;1     ALLNYLTAGLETPVHTLANGVSYGQGIIMEVILTFSLLFTVYTTIVDPKKGILEGMGPLL 173
NtTIP4;2     ALLNYVTAGLETPVHTLANGVSYSQGIIMEVILTFSLLFTVYTTIVDPKKGVLEGMGPLL 173
NtTIP3;1     LLLRLATDGLRPLGFSVAAGVGNLNALVMEIVMTFGLVYTVYATAIDPRRGSLHTIAPLA 180
NtTIP3;2     LLLRLATDGLRPLGFSVAAGVGNLNALVMEIVMTFGLVYTVYATAIDPRRGSLHTIAPLA 180
NtTIP3;3     ALLRLATDGLRPLGFGVAAGVGNLNALVMEIVMTFGLVYTVYATAIDPKRGSLGIIAPLA 179
NtTIP3;4     ALLRLATDGLRPLGFGVAAGVGNLNALVMEIVMTFGLVYTVYATAIDPKRGSLGIIAPLA 179
NtTIP2;4     YLLKFVTGGLAVPIHGVAAGVGATEGVVMEIIITFALVYTVFATAVDPKKGTLGTIAPIA 176
NtTIP2;5     YLLKFVTGGLAVPIHGVAAGVGATEGVVMEIIITFALVYTVFATAVDPKKGTLGTIAPIA 176
NtTIP2;3     YLLKVVTGGLAVPIHSVAAGVGAVEGVVMEIIITFALVYTVYATAADPKKGSLGTIAPIA 176
NtTIP2;1     LLLKVVTGGLAVPIHNVAAGVGALEGVVMEIIITFALVYTVYATAADPKKGSLGTIAPIA 176
NtTIP2;2     LLLKVVTGGLAVPTHNVAAGVGALEGVVMEIIITFALVYTVYATAADPKKGSLGTIAPIA 176
NtTIP2;8     LLLKYVTNGLAVPTHGVAAGLNGLQGVVMEIIITFALVYTVYATAADPKKGSLGTIAPIA 176
NtTIP2;9     LLLKYVTNGLAVPTHGVAAGLNGFQGVVMEIIITFALVYTVYATAADPKKGSLGTIAPIA 176
NtTIP2;10    LLLKYVTNGLAVPTHGVAAGLNGFQGVVMEIIITFALVYTVYATAADPKKGSLGTIAPIA 176
NtTIP2;6     LLLKFVTGGLAVPTHGVAAGLTGFEGVVMEIVITFALVYTVYATAADPKNGSLGTIAPIA 176
NtTIP2;7     LLLKFVTGGLAVPTHGVAAGLTGFEGVVMEIVITFALVYTVYATAADPQKGSLGTIAPIA 176
NtTIP1;4     LLLKFATGGLSTGAFALCSGLSVWNALVFEIVMTFGLVYTVYATAIDPKKGELGVIAPIS 178
NtTIP1;3     FLLEFATGGMSTGAFALSAGVSVWNAFVFEIVMTFGLVYTVYATAVDPKKGDLGVIAPIA 178
NtTIP1;1     FLLEFATGGMSTGAFALSAGVSVWNAFVFEIVMTFGLVYTVYATAIDPKKGDLGVIAPIA 178
NtTIP1;2     FLLEFATGGMSTGAFALSAGVSVWNAFVFEIVMTFGLVYTVYATAIDPKKGDLGVIAPIA 178
NtTIP1;5     VLLKFATGGLETSAFALSTGVTPWNAVVFEIVMTFGLVYTVYATAIDPKRGNLGIIAPIA 178
NtTIP1;6     VLLKFATGGLETSAFALSTGVTPWNAVVFEIVMTFGLVYTVYATAVDPKRGNLGIIAPIA 178
NtTIP1;7     FLLKFATGGLP--VFGLEIGAKIWNALVFEIVMTFGLVYTVYATAIDPKKGSLGTIAPIA 176
NtTIP1;8     LLLKFTTGGLEIGAFGLSDGVGVGNALVLEIVMTFGLVYTVYATAVDPNKGSLGTIAPIA 178
NtTIP1;9     FLLKFTTGGLEIGTFGLSDGVGVGNALVLEIVMTFGLVYTVYATAVDPKKGSLGTIAPIA 178
              :*.  .  :    . :  .     . ::* ::**.*::**:::  **..     :.*:
```

```
               TM5   H5       LE1  LE2P2 P3          P4P5   TM6
NtTIP5;1      IGLIVGANVMASGPFTGGSMNPAYSFGSAVVKGSFGNQAVYWVGPFIGAAIAGILYDNVV  232
NtTIP5;2      IGLIVGANVMASGPFTGGSMNPAYSFGSAVVKGSFGNQAVYWVGPFIGAAIAGILYDNVV  232
NtTIP4;1      TGLVVGANIMAGGPFSGASMNPARSFGPAFVSGIWTDHWVYWVGPLIGGGLAGFICENFF  233
NtTIP4;2      TGLVVGANIMAGGPFSGASMNPARSFGPAFVSGIWTDHWVYWVGPLIGGGLAGFICENFF  233
NtTIP3;1      IAFIVGANVLVGGPFEGGSMNPARAFGPALVGWRWRNHWIYWLGPFVGAALAGLIYEYGI  240
NtTIP3;2      IAFIVGANVLVGGPFEGGSMNPARAFGPALVGWRWRNHWIYWLGPFVGAALAGLIYEYGI  240
NtTIP3;3      IAFIVGANIFVGGPFEGASMNPARAFGPALVGWRWRNHWIYWLGPFIGAAIAGLIYEFGL  239
NtTIP3;4      IAFIVGANIFVGGPFEGASMNPARAFGPALVGWRWRNHWIYWLGPFIGAAIAGLIYEFGL  239
NtTIP2;4      IGFIVGANILAAGPFSGGSMNPARSFGPAVASGNFAGNWIYWVGPLVGGGLAGLTYSNVF  236
NtTIP2;5      IGFIVGANILAAGPFSGGSMNPARSFGPAVASGNFAGNWIYWVGPLVGGGLAGLTYSNVF  236
NtTIP2;3      IGFIVGANILAAGPFSGGSMNPARSFGPAVASGNFAGHWIYWVGPLVGGGLAGLIYSNVF  236
NtTIP2;1      IGFIVGANILAAGPFSGGSMNPARSFGPAVASGDFTNNWIYWAGPLVGGGLAGLTYSNVF  236
NtTIP2;2      IGFIVGANILAAGPFSGGSMNPARSFGPAVASGDFTNNWIYWAGPLVGGGLAGLIYSNVF  236
NtTIP2;8      IGFIVGANILAAGPFSGGSMNPARSFGPAVVAGDFSQNWIYWAGPLIGGGLAGFIYGDVF  236
NtTIP2;9      IGFIVGANILAAGPFSGGSMNPARSFGPAVVAGDFSQNWIYWAGPLIGGGLAGFIYGDVF  236
NtTIP2;10     IGFIVGANILAAGPFSGGSMNPARSFGPAVVAGDFSQNWIYWAGPLIGGGLAGFIYGDVF  236
NtTIP2;6      IGFIVGANILAAGPFSGGSMNPARSFGPAVVSGDFSQNWIYWVGPLIGGGLAGLIYGDVF  236
NtTIP2;7      IGFIVGANILAAGPFSGGSMNPARSFGPAVVSGDFSQNWIYWVGPLIGGGLAGLIYGDVF  236
NtTIP1;4      IGFIVGANILAGGPFDGASMNPAVSFGPAFVSWTWTHQWVYWAGPIIGAGLAGVVYELFF  238
NtTIP1;3      IGFIVGANILAGGAFTGASMNPAVSFGPALVSWTWTHQWVYWAGPLVGGGIAGVVYELIF  238
NtTIP1;1      IGFIVGANILAGGAFTGASMNPAVSFGPALVSWTWTHQWVYWAGPLVGGGIAGVVYELIF  238
NtTIP1;2      IGFIVGANILAGGAFTGASMNPAVSFGPALVSWTWTHQWVYWAGPLVGGGIAGVVYELIF  238
NtTIP1;5      IGFIVGANILAGGAFDGASMNPAVSFGPAVVSWTWNSHWVYWLGPFVGAAIAALVYEIIF  238
NtTIP1;6      IGFIVGANILAGGAFDGASMNPAVSFGPAVVSWTWNSHWVYWLGPFVGAATAALVYEIIF  238
NtTIP1;7      IGFIVGANILAGGAFDGASMNPAVSFGPAVVGWSRNNHWVYWAGPIIGGGLAGFVYEFFI  236
NtTIP1;8      IGFIVGANILAGGAFDGASMNPAVSFGPAVVSWSWANHWVYWAGPLIGGGLAGLVYEFFF  238
NtTIP1;9      IGFIVGANILAGGAFDGASMNPAVSFGPAVVSWSWNNHWVYWAGPLIGGGLAGLIYEFFF  238
               .::****::..* * *.***** :** *..      : :** **::*.. *..     .

NtTIP5;1      FPPQATESLR---GIGGGIAV      250
NtTIP5;2      FPSQATESLR---GIGGGIAV      250
NtTIP4;1      IVRT-------HVPLPSDESF      247
NtTIP4;2      IVRT-------HVPLPSDESF      247
NtTIP3;1      IQHEAVPRPTTHQPLAPEDY-      260
NtTIP3;2      IQHEAVPRPTTHQPLAPEDY-      260
NtTIP3;3      IQSEIAPIHTHHQPLAPEDY-      259
NtTIP3;4      IQSEIAPIHTHHQPLAPEDY-      259
NtTIP2;4      MNYD-------HAPLVSEF--      248
NtTIP2;5      MNYD-------HAPLVSEF--      248
NtTIP2;3      MNHD-------HAPLSTDF--      248
NtTIP2;1      MQNE-------HAPISSDF--      248
NtTIP2;2      MQNE-------HAPLSSDF--      248
NtTIP2;8      IGCH------TPLPTSEDYA-      250
NtTIP2;9      IGCH------TPLPTSEDYA-      250
NtTIP2;10     IGCH------TPLPTSEDYA-      250
```

```
NtTIP2;6       IGSH------DPLPVSEDYA-      250
NtTIP2;7       IGSH------DPLPVSEDYA-      250
NtTIP1;4       I-NH------SHEQVPTAEY-      251
NtTIP1;3       I-NH------SHEPLPSGDF-      251
NtTIP1;1       I-NH------SHEPLPSGDF-      251
NtTIP1;2       I-NH------SHEPLPSGDF-      251
NtTIP1;5       IGDN------THEQLPTADY-      252
NtTIP1;6       IGDN------THEQLPTADY-      252
NtTIP1;7       S--Q------TYEQLPPAEY-      248
NtTIP1;8       I-NQ------THEPLPQ----      248
NtTIP1;9       I-NQ------THEPLPQ----      248
```

Figure S3. Multiple sequence alignment of NtNIPs. The amino acid sequences were aligned using the Clustal Omega server. The two conserved NPA motifs are shown in yellow, the residues at H2, H5, LE1, and LE2 of the ar/R filter are in green, FPs (P1-P5) are in cyan, TM are shaded with grey background.

```
NtNIP7;1      ----------------------------------------------------MIMKL---   5
NtNIP7;2      ----------------------------------------------------MIMKL---   5
NtNIP8;1      ------------------------------------------------------------   0
NtNIP8;2      ------------------------------------------------------------   0
NtNIP6;1      MILSLLFIVSFGKQIWWHGQLKYLVQNGMDAEDGTSAPSTPATPGTPGAPLFGGFKHER-   59
NtNIP6;2      ------------------MLHLNSKCKGMDPEDGVSSPSTPATPGTPGAPLFGGFKHER-   41
NtNIP5;3      ------------------------------MQV----------------LAFGCS--NMG   12
NtNIP5;1      ------------------------------MPEFESPVSAPATPGTPT-PLFSSIRVDSM   29
NtNIP5;2      ------------------------------MPEFESPVSAPATPGTPT-PLFSSIRVDSM   29
NtNIP2;1      ------------------------------------------------------------   0
NtNIP3;1      ------------------------------------------------------------   0
NtNIP3;2      ------------------------------------------------------------   0
NtNIP1;1      ------------------------------------------------------------   0
NtNIP1;2      ------------------------------------------------------MKK---   3
NtNIP4;5      ------------------------------------------------------------   0
NtNIP4;6      ------------------------------------------------------------   0
NtNIP4;3      ------------------------------------------------------------   0
NtNIP4;4      ------------------------------------------------------------   0
NtNIP4;1      ------------------------------------------------------------   0
NtNIP4;2      ------------------------------------------------------------   0


NtNIP7;1      PSTENRL--------SLEFPAD-------ASTSEQST--YDQETT-------------SN   35
NtNIP7;2      LSSENRL--------SLEFPAD-------ASTSEQST--YDKETTSHSTSSNEEMLMKRN   48
NtNIP8;1      -----------------------MASITSIVSTNSSK--IGIVADF---SNMEE--GKHG   30
NtNIP8;2      -----------------------MASITSLVSTNSSK--NGIVADF---SSMEE--GKHG   30
NtNIP6;1      NSNGRNSLLKSLKCFS--V----------------------------------------E   77
NtNIP6;2      NSNGRNSLLKSLKCFS--V----------------------------------------E   59
NtNIP5;3      HSSHVSFRLPRTRCLP--HSQELIVILP-TSTSN---------QEVVSSSLPKS--KVRS   58
NtNIP5;1      ESNYDRKSMPRCKCLP--LDAP--------------------------------------   49
NtNIP5;2      GSNYDRKSMPRCKCLP--LDAP--------------------------------------   49
NtNIP2;1      -----------------------MESERGNSTENKKP------NELVSVENPKS--NL--   27
NtNIP3;1      --------MEEL------------SVVDGIRATSLRINSYPSPAESNTTATTPQ--K---   35
NtNIP3;2      --------MEDL------------PVVDGIRATSLRINSYPSPAESNTTATTSQ--K---   35
NtNIP1;1      ------------------MGDLQTAEANGNHASVSLNIRDNDMNSNNKTSAHED--SS--   38
NtNIP1;2      KTKLNKYQLEDLRVKSIDLSEKKIAEANGNHASVSLNIRDNDMNSNNKTSAHED--SS--   59
NtNIP4;5      -----------------------------MSKKDIKAVEEGNCSG-YKQHANEE--DS--   26
NtNIP4;6      -----------------------------MSKKDIKAVEEGDCSG-YKQHANEE--DS--   26
NtNIP4;3      -----------------------MAKKDGNREEEISQMEEGNI-H--SASKSDS--NV--   30
NtNIP4;4      -----------------------MAKKDGNREEEISQMEEGNITH--SASQSDS--NV--   31
NtNIP4;1      ----------------------MAANTEGSREDEISKLEEGVHADNICASQSNT--SI--   34
NtNIP4;2      ----------------------MAAQIEGIREDEISKLEEGVRADNICASQSNT--SI--   34
```

```
                                                   TM1
NtNIP7;1      FWNSPLG----------LINPTLIRMVLAEALGTFMLMFCICGIMASMEIMGVRAGLMEY  85
NtNIP7;2      FWNSPLG----------LINPTLLHMVLAEALGTFMLMFCICGIMASMEIMGVRVGLMEY  98
NtNIP8;1      SFRSPMKEF----SPRAATFMSSFQKIIAELVGTYIFIFVGCGSALVDRE--RTLTIVGI  84
NtNIP8;2      VFRTPMKEL----SPGAATFMSAFQKIVAELVGTYIFIFVGCGSALVDRE--RTLTIGGI  84
NtNIP6;1      AWASEEGSLPPVSCALPPPFVSLARKVGAEFIGTMILIFAGTATAIVNQKTQGSETLIGL  137
NtNIP6;2      AWASEEGSLPPVSCALPPPFVSLARKVGAEFIGTMILIFAGTATAIVNQKTQGSETLIGL  119
NtNIP5;3      SWR--EGC----RGSIWKQFLYPRVGLGAEFVGTFILIFAATAGPIVNQKYNGAESLIGN  112
NtNIP5;1      TWGTPHTC----LSDFPAPDVSLTRKLGAEFVGTFILIFAATAGPIVNQKYSGVESLIGN  105
NtNIP5;2      TWGTPHTC----LSDFPAPDVSLTRKLGAEFVGTFILIFAATAGPIVNQKYNGAESLIGN  105
NtNIP2;1      SFR------YILIFFQEHYFPGFLKKVIAEVIATYLLVFVTCGAAAISASDEHKVSRLGA  81
NtNIP3;1      -------------HSKCFISVHFVQKLIAELVGTYMLIFAGCAAIVLNINKDNVVTLPGI  82
NtNIP3;2      -------------HSKCLISVHFVQKLIAELVGTYMLIFAGCAAIVLNINKDNVVTLPGI  82
NtNIP1;1      S------------SCC-FVTVPFIQKIIAETLGTYFLIFAGCGSVAVNADK-GMVTFPGI  84
NtNIP1;2      S------------SCC-FVTVPFIQKIIAETLGTYFLIFAGCGSVAVNADK-GMVTFPGI  105
NtNIP4;5      S------------LCTSPEVVIIIQKVIAEAIGTYFLIFVGCGAVAVNKTY-GSVTFPGI  73
NtNIP4;6      S------------LCTSPEVVIIIQKVIAEAIGTYFLIFVGCGAVAVNKTY-GSVTFPGI  73
NtNIP4;3      G------------FCSSVSVVVILQKLIAEAIGTYFVIFAGCGSVAVNKIY-GSVTFPGI  77
NtNIP4;4      G------------FCSSLTVVVIAQKLIAEAIGTYFVIFAGCGSVAVNKIY-GSVTFPGI  78
NtNIP4;1      G------------FCSSPSVVAVAQKLIAEAIGTYFIIFAGCGSVAVNKLYDGSITFPGI  82
NtNIP4;2      G------------FCSSPSVVAVAQKLIAEAIGTYFIIFAGCGSVAVNKLYDGSITFPGI  82
                                  : ** :.* :.:*   .

                 H2  TM2
NtNIP7;1      AATAALTVVVVVFSIGPISGAHINPAVTLAFAAVGHFPWS--------------------  125
NtNIP7;2      AATAALTVVVVVFSIGPISGAHINPAVTLAFAAVGHFPWS--------------------  138
NtNIP8;1      AMAWGLSLMGLIYTLGHVSGAHFNPAVTIAFAAARKLPLLH-------------------  125
NtNIP8;2      AMAWGLSLMGLIYTLGHFSGAHFNPAVTIALAAARKLPLLHPQVRMRGAYPAITDAKHLA  144
NtNIP6;1      AASSGLAVMIVILSTGHISGAHLNPAVTIAFAALKHFPWK--------------------  177
NtNIP6;2      AASTGLAVMIVILSTGHISGAHLNPAVTIAFAALKHFPWK--------------------  159
NtNIP5;3      AACSGLAVMIVILSTGHISGAHLNPSLTIAFAALRHFPWV--------------------  152
NtNIP5;1      AACAGLAVMIVILSTGHISGAHLNPSLTIAFAALRHFPWV--------------------  145
NtNIP5;2      AACSGLAVMIVILSTGHISGAHLNPSLTIAFAALRHFPWV--------------------  145
NtNIP2;1      SVAGGLIVTVMIYAVGHISGAHMNPAVTFAFAAVRHFPWR--------------------  121
NtNIP3;1      ASVWGLVVMVLIYSVGHVSGAHFNPAVTIAFASCKRFPWN--------------------  122
NtNIP3;2      ASVWGLVVMVLIYSVGHVSGAHFNPAVTIAFASCKRFPWN--------------------  122
NtNIP1;1      SIVWGLVVMVMVYSVGHISGAHFNPAVTISFATCKRFPWK--------------------  124
NtNIP1;2      SIVWGLVVMVMVYSVGHISGAHFNPAVTISFATCKRFPWK--------------------  145
NtNIP4;5      CVAWGLIVMVMVYAVGHISGAHFNPAVTIAFAIFRHFPFK--------------------  113
NtNIP4;6      CVAWGLIVMVMVYSVGHISGAHFNPAVTIAFAIFRHFPFK--------------------  113
NtNIP4;3      CVTWGLIVMVMVYTVGHISGAHFNPAVTITFSILGHFPWK--------------------  117
NtNIP4;4      CVTWGLIVMVMVYTVGHISGAHFNPAVTITFTIFGRFPWK--------------------  118
NtNIP4;1      CVTWGLIVMVMIYSLGHVSGGHFNPAVTIAFTIFRRFSWK--------------------  122
NtNIP4;2      CVTWGLIVMVMIYSLGHVSGAHFNPAVTIAFTIFRRFSWK--------------------  122
              .    .* :  :: : * .**.*:**::*::::   ::
```

```
                                                              TM3             P1
NtNIP7;1      -----------------------------KVPFYVVAQVGGSILATYTGKLVYGLK-AEF   155
NtNIP7;2      -----------------------------KVPFYVVAQVGGSILATYTGKLVYGLK-AEF   168
NtNIP8;1      ------------------------------VPMYVFPQFLGSTLACLTLRVLFNHQGDIL   155
NtNIP8;2      HLRTRKCEDPVHRSGGMRRCEGRRYEYLPQVPMYVFPQFLGSTLACLTLSVLFNHQGDIL   204
NtNIP6;1      -----------------------------NVPVYIGAQIIASFCAAFTLKVVLHPI-MGG   207
NtNIP6;2      -----------------------------NVPVYIGAQIIASFCAAFTLKVVLHPI-MGG   189
NtNIP5;3      -----------------------------QVPAYVAAQVSASVCASFALKGVFHPF-MSG   182
NtNIP5;1      -----------------------------QVPAYVAAQVSASVCASFALKGVFHPF-MSG   175
NtNIP5;2      -----------------------------QVPAYVAAQVSASVCASFALKGVFHPF-MSG   175
NtNIP2;1      -----------------------------QVPPYAAAQLTGATSAAFTLRVLLHPI-KHV   151
NtNIP3;1      -----------------------------QVPAYILVQVIGSTLASGSLRLIFNGKEDQF   153
NtNIP3;2      -----------------------------QVPAYILVQVIGSTLASGSLRLIFNGKEDQF   153
NtNIP1;1      -----------------------------QVPAYVAAQVIGSTLASGTLRLIFNGKHDHF   155
NtNIP1;2      -----------------------------QVPAYVAAQVIGSTLASGTLRLIFNGKHDHF   176
NtNIP4;5      -----------------------------QVPLYILAQLVGAILGSGTLYLLLDLKSEAF   144
NtNIP4;6      -----------------------------QVPLYILAQLVGSILGSGTLYLLLDLKSEAF   144
NtNIP4;3      -----------------------------QVPLYIIAQLMGSILASGTLALLFDVTPQAY   148
NtNIP4;4      -----------------------------QVPLYIIAQLMGSILASGTLALLFDVTPQAY   149
NtNIP4;1      -----------------------------LAPLYIIAQVTGSILASGTLALLLDVTSTSY   153
NtNIP4;2      -----------------------------LAPLYIIAQLTGSILSSGTLALLLDVTSTSY   153
                                              .* *   *. .:  .  :   :

                                 TM4                               TM5  H5
NtNIP7;1      VIT---RPLHGCTSAFFVELLATFIVLFLTASLTNDPQSTGPLSGFVVGVAIGLAVLISG   212
NtNIP7;2      VIT---RPLHGSTSAFFVELLATFIVLFLTASLTNDPQSTGPLSGFVVGVAIGLAVLISG   225
NtNIP8;1      PTLTQYKNPVTDFEAITWEFIMTLILMFVICGAATDDRASKELAGVAIGVTLLFEVLIAG   215
NtNIP8;2      PTLTQYKNPVTDFEAITWEFIMTLILMFVICGAATDDRASKEQAGVAIGVTLLFEVLIAG   264
NtNIP6;1      GVT---VPSGSYLQAFALEFIISFNLMFVITAVATDTRAVGELAGIAVGATVMLNILIAG   264
NtNIP6;2      GVT---VPSGSYLQAFALEFIISFNLMFVVTAVATDTRAVGELAGIAVGATVMLNILIAG   246
NtNIP5;3      GVT---VPSVNTGQAFALEFLITFNLLFVVTAVATDTRAVGELAGIAVGATVMLNILVAG   239
NtNIP5;1      GVT---VPSVNTGQAFALEFLITFNLLFVVTAVATDTRAVGELAGIAVGATVMLNILVAG   232
NtNIP5;2      GVT---VPSVNTGQAFALEFLITFNLLFVVTAVATDTRAVGELAGIAVGATVMLNILVAG   232
NtNIP2;1      GTT---TPSGSDIQALIMEIVVTFSMMFITSAVATDTKAIGELAGMAVGSAVCITSILAG   208
NtNIP3;1      VGT---VPAGTNMQALVLEFIATFYLMFVISGVATDDRAMKHLSGVAIGATVSLDILFSG   210
NtNIP3;2      VGT---VPAGTNMQAFILEFIATFYLMFVISGVATDDRAMKHFSGVAIGATVSLDILFSG   210
NtNIP1;1      LGT---SPSGSDIQSLVLEFIITFYLMFVVSGVATDNRAIGELAGLAVGATVLLNVMFAG   212
NtNIP1;2      LGT---SPSGSDIQSLVLEFIITFYLMFVVSGVATDNRAIGELAGLAVGATVLLNVMFAG   233
NtNIP4;5      FGT---APVGSNVQSLVLEFIISYLLMFVISGVATDNRSIGELAGIAIGMTILLNVLVAG   201
NtNIP4;6      FGT---APVGSNVQSLVLEFIISYLLMFVISGVATDNRSIGELAGIAIGMTILLNVLVAG   201
NtNIP4;3      FGT---VPVGSNGQSLAIEIIISFLLMFVISGVATDDRAIGQVAGIAVGMTITLNVFVAG   205
NtNIP4;4      FGT---VPVESNGQSLAIEIIISFLLMFVISGVATDDRAIGQVAGIAVGMTITLNVFVAG   206
NtNIP4;1      FGT---VPVGSNSQSLAMEIIISFLLMFVVCGVSTDDRAIGELAGLAVGMTITLNVFVAG   210
NtNIP4;2      FGT---VPVGSNGQSLAMEIIISFLLMFVVCGVSTDVRAIGELSGLAVGMTITLNVFVAG   210
                       .::  *:: :  ::*:  . :.* ::    :*..:* :: :  :.:*
```

```
                    LE1  LE2P2 P3          P4P5   TM6
NtNIP7;1        ---PISGGSMNPARSLGPAIVSLKFGGLWIYVVAPILGAVAGVVLYRLLRLQGWSCKPNS   269
NtNIP7;2        ---PISGGSMNPARSLGPAIVSWKFGGLWIYVVAPILGAVAGVVLYRLLRLQGWSCKPNS   282
NtNIP8;1        ---PITGASMNPARSLGPAIVSGVYKNQWIFVIAPILGAMTATGIYSLLRQPKQEMLMSK   272
NtNIP8;2        ---PITGASMNPARSLGPAIVSGVYKNQWVFVIAPILGAMTATGIYSLLRQPK-------   314
NtNIP6;1        ---ETTGASMNPVRTLGPAVAAGNYKAIWIYLTAPILGALAGAGIYSAVKLPNEDDNNHG   321
NtNIP6;2        ---ETTGASMNPVRTLGPAVAAGNYKAIWIYLTAPILGALAGAGVYSAVKLPNEDDNNHG   303
NtNIP5;3        ---PSSGASMNPVRTLGPAVAAGNYKSLWIYIVAPTLGALAGAAVYTLVKLRGDDSSE--   294
NtNIP5;1        ---PSSGASMNPVRTLGPAVAAGNYKSLWIYLVAPTLGALAGAAVYTLVKLRGDDSTE--   287
NtNIP5;2        ---PSSGASMNPVRTLGPAVAAGNYKSLWIYIVAPTLGALAGAAVYTLVKLRGDDSSE--   287
NtNIP2;1        ---PVSGGSMNPARTIGPAMASNDYRGIWVYIIGPVCGTLLGAWSYNFIRVTDKPVHAIA   265
NtNIP3;1        ---PLTGASMNPARSLGPAIVTGHYKGLWIYIIGPTLGAIFGAWTYNLMRLTNKSWGESI   267
NtNIP3;2        ---PLTGASMNPARSLGPAIVTGHYKGLWIYIIGPTLGAIFGAWTYNLMRLTNKSWGEAV   267
NtNIP1;1        ---PISGASMNPARSLGPAIVWSHYRGIWVYMLGPTAGAISGAWVYNIIRFTDKPLREIT   269
NtNIP1;2        ---PISGASMNPARSLGPAIVWSHYRSIWVYMLGPTAGAISGAWVYNIIRFTDKPLREIT   290
NtNIP4;5        ---PVSGASMNPARSIGPAIVMHEYKGLWVYIVGPILGTIVGAFTYNLIRFTEKPLKELT   258
NtNIP4;6        ---PVSGASMNPARSIGPAIVMHEYKGLWVYIVGPILGTIVGAFTYNLIRFTEKPLRELT   258
NtNIP4;3        ---PISGASMNPARSIGPAIVKHVYTGLWVYIVGPIIGTLAGAFVYNLIRSTDKPLRELA   262
NtNIP4;4        ---PISGASMNPARSIGPAIVKHVYTGLWVYIVGPIIGTLAGAFVYNLIRSTDKPLRELA   263
NtNIP4;1        GCRPISGASMNPARSIGPAIVKQVYKGLWVYIIGPVIGTLAGAFVYNLIRFTNKPLLQLV   270
NtNIP4;2        ---PISGASMNPARSIGPAIVKQVYKGLWVYIIGPVIGTLAGAFVYNLIRFTNKPLLQLV   267
                     :*.****.*::***:.   :   *::: .*  *:: ..  *  ::

NtNIP7;1        TPTTTHQHNPL-------------------------------------------------   280
NtNIP7;2        TPTTTHQHNPL-------------------------------------------------   293
NtNIP8;1        ALQSI-------------------------------------------------------   277
NtNIP8;2        ------------------------------------------------------------   314
NtNIP6;1        KPS-VEHSFRR-------------------------------------------------   331
NtNIP6;2        KPS-LEHSFRR-------------------------------------------------   313
NtNIP5;3        TPR-QVRSFRR-------------------------------------------------   304
NtNIP5;1        TPR-QVRSFRR-------------------------------------------------   297
NtNIP5;2        TPR-QVRSFRR-------------------------------------------------   297
NtNIP2;1        PGQSFSFKLRRMKSNDEEQGV---------------------------------------   286
NtNIP3;1        KEISDSQKIIEVSSKDKVICKCSEGWTCIVTKTEEPEAGNIFFDCGDEGCLCIIDETNTL   327
NtNIP3;2        KEISESQKVIEVSSKDKVICKCSEGWTCIVTKTEEPEAHNIFFECGDEGCVCIIDETHTL   327
NtNIP1;1        KSGSFLKSIRSSKSLRSST-----------------------------------------   288
NtNIP1;2        KSGSFLKSIRSST-----------------------------------------------   303
NtNIP4;5        KSSTFLKSMSRSHA----------------------------------------------   272
NtNIP4;6        KSSTFLKSMSRSHT----------------------------------------------   272
NtNIP4;3        KSASSLRS----------------------------------------------------   270
NtNIP4;4        KTASSLRS----------------------------------------------------   271
NtNIP4;1        KSRSFLPKLRE-------------------------------------------------   281
NtNIP4;2        KSRSFLRS----------------------------------------------------   275
```

NtNIP7;1	--------------------	280
NtNIP7;2	--------------------	293
NtNIP8;1	--------------------	277
NtNIP8;2	--------------------	314
NtNIP6;1	--------------------	331
NtNIP6;2	--------------------	313
NtNIP5;3	--------------------	304
NtNIP5;1	--------------------	297
NtNIP5;2	--------------------	297
NtNIP2;1	--------------------	286
NtNIP3;1	KKHVLVYDKTKRRKGYKMYI	347
NtNIP3;2	KKHVYVYDKTKRRRSYKMYI	347
NtNIP1;1	--------------------	288
NtNIP1;2	--------------------	303
NtNIP4;5	--------------------	272
NtNIP4;6	--------------------	272
NtNIP4;3	--------------------	270
NtNIP4;4	--------------------	271
NtNIP4;1	--------------------	281
NtNIP4;2	--------------------	275

Figure S4. Multiple sequence alignment of NtSIPs. The amino acid sequences were aligned using the Clustal Omega server. The two conserved NPA motifs are shown in yellow, the residues at H2, H5, LE1, and LE2 of the ar/R filter are in green, FPs (P1-P5) are in cyan, TM are shaded with grey background.

```
                                      TM1                    H2 TM2
NtSIP2;1      MGVSRRRLLVSDFIMSFMWVWSSVLIKMFVHTM-LGYGAHDLKGEI-LKHSISVINMFFF  58
NtSIP1;1      MGV--IKAAIGDGALTFLWVLCSSCIGVSTYLVATTFGVVNEMGSLFITTLVVFLIFLVF  58
NtSIP1;2      MGA--VKAAVADFVLTSMWVFCSSTLGVFTYLIASAFGIAQGITTLFITTVLLFILFFVF  58
              **.   :  :.*  :: :** .*  : : .: :   :*  :    : :.  : .: ::.*

                                                    TM3             P1
NtSIP2;1      AFLAKATKGGAYNPLTILSGAISGDLSNFLFTVGARIPAQVFGSITGVRLIIA----AFP  114
NtSIP1;1      GFLGDALGGAAFNPTANAAFYAAGLGHDSLVSAALRCPAQVAGAVAGSLAIMELMPKQYH  118
NtSIP1;2      GIIGDALGGAAFNPAGTAAFYAAGVGTDSLFSVAARFPAQAAGAVAGALAILEVIPTQYK  118
              .::..*  *.*:**    :   :*   : *.:.. * ***. *:::*    *:     :

                                        TM4                    TM5   H5
NtSIP2;1      NIGRGPRLTIDIHRGALIEGCLTFAIVTISLGLSRRSRASTLMKTWISSLSKLTLHILGS  174
NtSIP1;1      HMLEGPALKVDVQAGAIAEGVLTFIITFMVFVIVLRGPKSALLKNWLLTLVTVPLVVAGS  178
NtSIP1;2      HMLGGPSLKVDLHNGAIAEGILTFTMTFLVFLIVLRGPRNALLKNWLLAMSTVTMVVTGS  178
              ::  ** *.:*:: **: ** *** :. : : :  *.  .:*:*. *: :: .: : **

                 LE1  LE2P2 P3             P4P5  TM6
NtSIP2;1      DLTGGCMNPASVMGWAYARGDHITKEHIHVYWLAPIQATLLAVWTFNLLVSPPKDEREKM  234
NtSIP1;1      NYTGPSMNPANVVPTNQ-------------LWTLPCISKLKMTAAKKCTFSSISVLEKRQ  225
NtSIP1;2      KYTGPSMNPANAFGWAYINNWHNTWEQFYVYWICPFVGAIMAAWTFRALFPPPVKQKPQK  238
              . ** .****...                  *  *  . :  . : .  .      . :

NtSIP2;1      IEKKSE-------      240
NtSIP1;1      VKKGANRMCHLSF      238
NtSIP1;2      AKKN---------      242
               :*
```

Figure S5. Multiple sequence alignment of NtXIPs. The amino acid sequences were aligned using the Clustal Omega server. The two conserved NPA motifs are shown in yellow, the residues at H2, H5, LE1, and LE2 of the ar/R filter are in green, FPs (P1-P5) are in cyan, TM are shaded with grey background.

```
NtXIP1;1α      MASNASHVLGDEESQLSGGSNRVQPFSSTPK-KNIDDEGKKHTSLTVAQRLGISDFFSLD  59
NtXIP1;1β      MASNASHVLGDEESQLSGGSNRVQPFSSTPKNRNIDDEGKKHTSLTVAQRLGISDFFSLD  60
NtXIP1;2α      MASNTSHVLGDEESQLSGGSNRVQPFSSTPKNRNIDDEGKKHTSLTVAQRLGISDFFSLD  60
NtXIP1;2β      MASNTSHVLGDEESQLSGGSNRVQPFSSTPK-KNIDDEGKKHTSLTVAQRLGISDFFSLD  59
NtXIP2;1       MAATTRDALDDEE-------------TQFSSIPKIDEENKKKSPLTLSQRLGLPDFFSPD  47
NtXIP2;2       ML-----WVMKKP-------------NSPLYQSKIDEENKKKAPLTLSQRLGLPDFVSPD  42
               *       : .:              ..     :**:*.**:: **::****: **.* *

                        TM1                                H2     TM2
NtXIP1;1α      VWRASVGELLGSAVLVFMLDTIVISTFESDVKMPNLIMSILIAIVITILLLAVVPVSGGH  119
NtXIP1;1β      VWRASVGELLGSAVLVFMLDTIVISTFESDVKMPNLIMSILIAIVITILLLAVVPVSGGH  120
NtXIP1;2α      VWRASMGELLGSAVLVFMLDTIVISTFESDVKMPNLIMSILIAIVITILLLAVVPVSGGH  120
NtXIP1;2β      VWRASMGELLGSAVLVFMLDTIVISTFESDVKMPNLIMSILIAIVITILLLAVVPVSGGH  119
NtXIP2;1       VWRASVGELLGTAVLVFMLDTIVISTLESDVKMPNLIMSILAAITITILLLAVFPVSGGH  107
NtXIP2;2       VWRASVGELLGTAVLVFMLDNIVISTLESDVKMPNLIMSILAAITITILLLAVFPVSGGH  102
               *****:*****:********.*****:************** **.********.******

                                           TM3             P1
NtXIP1;1α      INPVISFSAALVGIISMSRAIIYMVAQCVGAILGALALKAVVSSTIAQTFSLGGCTITVI  179
NtXIP1;1β      INPVISFSAALVGIISMSRAIIYMVAQCVGAILGALALKAVVSSTIAQTFSLGGCTITVI  180
NtXIP1;2α      INPVISFSAALVGIISMSRAIIYMVAQCVGAILGALALKAVVSSTIAQTFSLGGCTITVI  180
NtXIP1;2β      INPVISFSAALVGIISMSRAIIYMVAQCVGAILGALALKAVVSSTIAQTFSLGGCTITVI  179
NtXIP2;1       LNPVISCSAALVGIISMSRAIIYIVAQCIGATLGALALKAVVSSSIENTFSLGGCTLTVI  167
NtXIP2;2       LNPVISCSATLVGIISMSRAIIYIVAQCIGAALGALALKAVVSSSIEQTFSLGGCTLTVI  162
               :***** **:*************:****:** ************:* :********:***

NtXIP1;1α      APGPNGPITVGLEMAQALWLEIFCTFVFLFASIWMAYDHRQAKALGLVTVLSIVGIVLGL  239
NtXIP1;1β      APGPNGPITVGLEMAQALWLEIFCTFVFLFASIWMAYDHRQAKALGLVTVLSIVGIVLGL  240
NtXIP1;2α      APGPNGPITVGLETAQALWLEIFCSFVFLFASIWMAYDHRQAKALGLVTVLSIVGIVLGL  240
NtXIP1;2β      APGPNGPITVGLETAQALWLEIFCSFVFLFASIWMAYDHRQAKALGLVTVLSIVGIVLGL  239
NtXIP2;1       SSGPNGPITVGIETAQAFWLEIFCTFVFLFASVWMAYDHRQAKSLGLVTIMSIIGLVLGL  227
NtXIP2;2       LPGPNGPITVGLETAQAFWLEIFCTFVFLFASVWMAYDHRQAKSLGLVTVMSIVGLVLGL  222
                 *********:* ***:******:*******:**********:*****::**:*:****

                 TM5  H5       LE1  LE2P2 P3             P4P5  TM6
NtXIP1;1α      LVFISTTVTMKKGYAGAGMNPARCFGAAVVRGGHLWDGHWIFWVGPTIACVAFYVYTKII  299
NtXIP1;1β      LVFISTTVTMKKGYAGAGMNPARCFGAAVVRGGHLWDGHWIFWVGPTIACVAFYVYTKII  300
NtXIP1;2α      LVFISTTVTAKKGYAGAGMNPARCFGAAVVRGGHLWDGHWIFWVGPTIACVAFYVYTKII  300
NtXIP1;2β      LVFISTTVTAKKGYAGAGMNPARCFGAAVVRGGHLWDGHWIFWVGPTIACVAFYVYTKII  299
NtXIP2;1       LVFISTTVTGKKGYAGAGMNPARCFGAALVRGGNLGDGHWIFWVGPAIACWAFYLYTKII  287
NtXIP2;2       LVFISTTVTGKKGYAGAGMNPARCFGAALVRGGHLWNGHWIFWVGPAIACWAFYLYTKII  282
               ********* ******************:****:* :*********:*** ***:*****

NtXIP1;1α      PPKHFHADGYKYDFIGVVKASFGLHE  325
NtXIP1;1β      PPKHFHADGYKYDFIGVVKASFGLHE  326
```

```
NtXIP1;2α      PPQHFHADGYKYDFIGVVKASFGLHV 326
NtXIP1;2β      PPQHFHADGYKYDFIGVVKASFGLHV 325
NtXIP2;1       PLQHFHADGYKHDFFGVIKSLFGSDA 313
NtXIP2;2       PPQHFHADGYKHDFVGVIKALFGSDA 308
               * :********:**.**:*: ** .
```

Figure S6. Exon-intron organization of *NtAQP* genes. Yellow boxes represent the coding regions or exons, blue color represents the upstream/downstream, and black lines represent the introns (software GDS2;0).

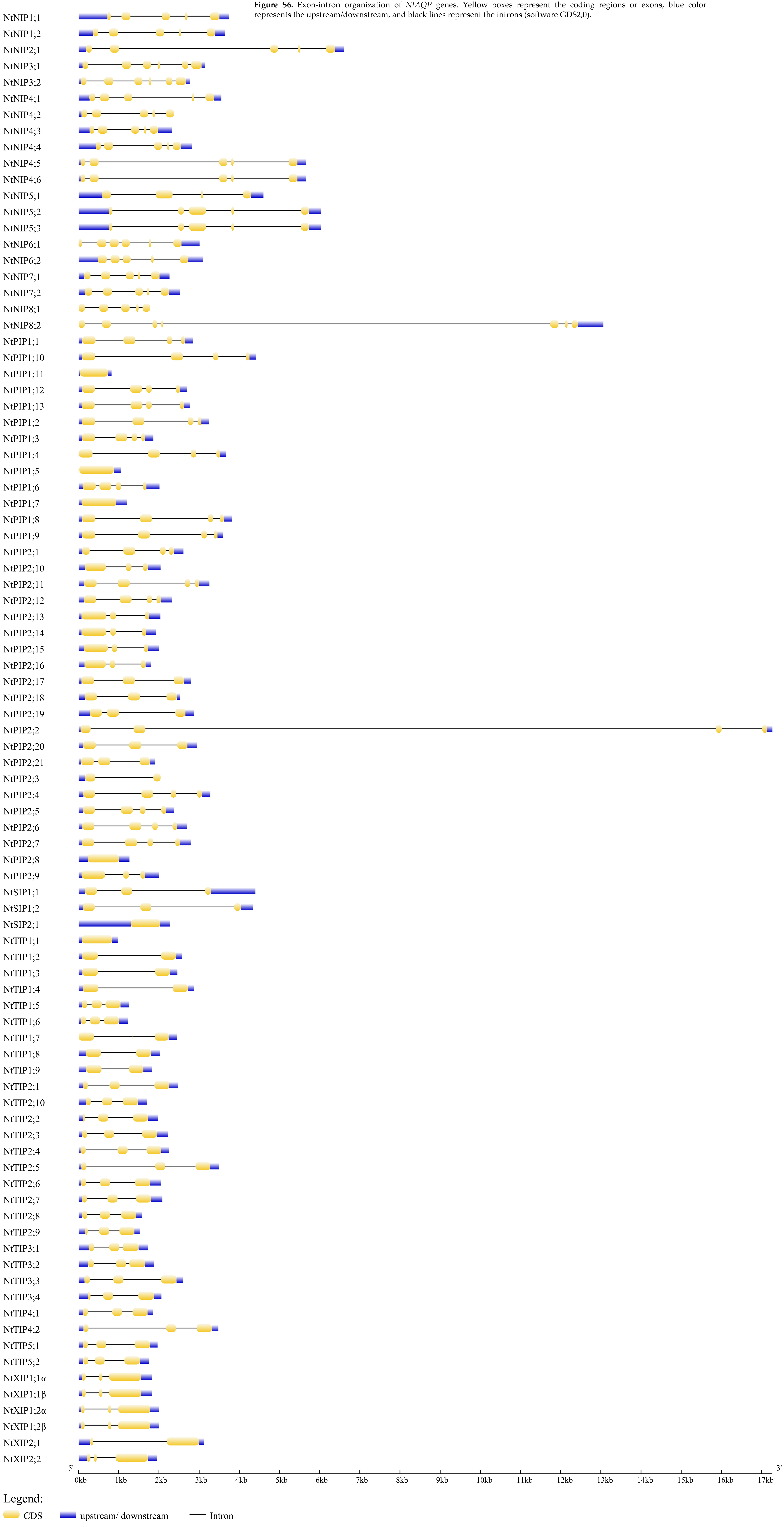

Table S1. Aquaporins in *N. tabacum*, *N. tomentosiformis*, and *N. sylvestris*

N. tabacum	*N. tomentosiformis*	*N. sylvestris*
**NtPIP1;1**	**NtoPIP1;1**	**NsPIP1;1**
**NtPIP1;2**	**NtoPIP1;2**	**NsPIP1;2**
**NtPIP1;3**	**NtoPIP1;3**	**NsPIP1;3**
**NtPIP1;4**	**NtoPIP1;4**	**NsPIP1;4**
**NtPIP1;5**	**NtoPIP1;5**	**NsPIP1;5**
**NtPIP1;6**	-	**NsPIP1;6**
**NtPIP1;7**	-	-
**NtPIP1;8**	-	-
**NtPIP1;9**	-	-
**NtPIP1;10**	-	-
**NtPIP1;11**	-	-
**NtPIP1;12**	-	-
**NtPIP1;13**	-	-
**NtPIP2;1**	**NtoPIP2;1**	**NsPIP2;1**
**NtPIP2;2**	**NtoPIP2;2**	**NsPIP2;2**
**NtPIP2;3**	**NtoPIP2;3**	**NsPIP2;3**
**NtPIP2;4**	**NtoPIP2;4**	**NsPIP2;4**
**NtPIP2;5**	**NtoPIP2;5**	**NsPIP2;5**
**NtPIP2;6**	**NtoPIP2;6**	**NsPIP2;6**
**NtPIP2;7**	**NtoPIP2;7**	**NsPIP2;7**
**NtPIP2;8**	**NtoPIP2;8**	**NsPIP2;8**
**NtPIP2;9**	**NtoPIP2;9**	**NsPIP2;9**
**NtPIP2;10**	**NtoPIP2;10**	**NsPIP2;10**
**NtPIP2;11**	**NtoPIP2;11**	-
**NtPIP2;12**	**NtoPIP2;12**	-
**NtPIP2;13**	-	-
**NtPIP2;14**	-	-
**NtPIP2;15**	-	-
**NtPIP2;16**	-	-
**NtPIP2;17**	-	-

NtPIP2;18	-	-
NtPIP2;19	-	-
NtPIP2;20	-	-
NtPIP2;21	-	-
NtTIP1;1	NtoTIP1;1	NsTIP1;1
NtTIP1;2	NtoTIP1;2	NsTIP1;2
NtTIP1;3	NtoTIP1;3	NsTIP1;3
NtTIP1;4	NtoTIP1;4	-
NtTIP1;5	-	-
NtTIP1;6	-	-
NtTIP1;7	-	-
NtTIP1;8	-	-
NtTIP1;9	-	-
NtTIP2;1	NtoTIP2;1	NsTIP2;1
NtTIP2;2	NtoTIP2;2	NsTIP2;2
NtTIP2;3	NtoTIP2;3	NsTIP2;3
NtTIP2;4	NtoTIP2;4	NsTIP2;4
NtTIP2;5	NtoTIP2;5	NsTIP2;5
NtTIP2;6	-	-
NtTIP2;7	-	-
NtTIP2;8	-	-
NtTIP2;9	-	-
NtTIP2;10	-	-
NtTIP3;1	NtoTIP3;1	NsTIP3;1
NtTIP3;2	NtoTIP3;2	NsTIP3;2
NtTIP3;3	-	-
NtTIP3;4	-	-
NtTIP4;1	NtoTIP4;1	NsTIP4;1
NtTIP4;2	-	-
NtTIP5;1	NtoTIP5;1	NsTIP5;1
NtTIP5;2	-	-
NtNIP1;1	NtoNIP1;1	NsNIP1;1
NtNIP1;2	NtoNIP1;2	NsNIP1;2
NtNIP2;1	NtoNIP2;1	NsNIP2;1
NtNIP3;1	NtoNIP3;1	NsNIP3;1

**NtNIP3;2**	**-**	**NsNIP3;2**
**NtNIP4;1**	**NtoNIP4;1**	**NsNIP3;3**
**NtNIP4;2**	**NtoNIP4;2**	**NsNIP4;1**
**NtNIP4;3**	**NtoNIP4;3**	**NsNIP4;2**
**NtNIP4;4**	**NtoNIP4;4**	**NsNIP4;3**
**NtNIP4;5**	**NtoNIP4;5**	**NsNIP4;4**
**NtNIP4;6**	**-**	**NsNIP4;5**
**NtNIP5;1**	**NtoNIP5;1**	**NsNIP5;1**
**NtNIP5;2**	**-**	**-**
**NtNIP5;3**	**-**	**-**
**NtNIP6;1**	**NtoNIP6;1**	**NsNIP6;1**
**NtNIP6;2**	**NtoNIP6;2**	**NsNIP6;2**
**NtNIP7;1**	**NtoNIP7;1**	**NsNIP7;1**
**NtNIP7;2**	**-**	**-**
**NtNIP8;1**	**-**	**NsNIP8;1**
**NtNIP8;2**	**-**	**-**
**NtSIP1;1**	-	-
**NtSIP1;2**	-	-
**NtSIP2;1**	**NtoSIP2;1**	**NsSIP2;1**
**NtXIP1;1α**	**NtoXIP1;1α**	**NsXIP1;1α**
**NtXIP1;1β**	**NtoXIP1;1β**	**NsXIP1;1β**
**NtXIP1;2α**	**NtoXIP1;2**	**NsXIP1;2**
**NtXIP1;2β**	**NtoXIP1;3**	**NsXIP1;3**
**NtXIP2;1**	**NtoXIP1;4**	**NsXIP2;1**
**NtXIP2;2**	**NtoXIP2;1α**	
	**NtoXIP2;1β**	
90	***51***	***50***

Table S2. List of AQPs in *Nicotiana tabacum* predicted to transport substrates based on SDPs in NPA regions, ar/R selectivity filter and FPs separately.

**NtAQPs**	**Ar/R (H2-H5-LE1-LE2)**		**LB (NPA region)**		**LE (NPA region)**		**FPs (P1-P5)**	
**Non-aqua**	Motif [hit]	isoforms	Motif [hit]	isoforms	Motif [hit]	isoforms	Motif [hit]	isoforms
**Boron**	[AGI][ISV][GA]R [4]	NtNIP2;1<br>NtNIP5;1<br>NtXIP1;1-2	SG[AG]H[ILM]NP[ASV][VLI][TS] [57]	NtPIP1;1-13<br>NtPIP2;1-21<br>NtTIP2;6-10<br>NtTIP3;1-4<br>NtTIP4;1-2<br>NtNIP2;1<br>NtNIP5;1-3<br>NtNIP6;1-2<br>NtNIP7;1-2<br>NtXIP1;1-2<br>NtXIP2;1-2	[GS][GA][SG]MNP[AV]R[STC][LF]G [29]	NtTIP2;1-10<br>NtTIP4;1-2<br>NtNIP1;1-2<br>NtNIP3;1-2<br>NtNIP5;1-3<br>NtNIP6;1-2<br>NtNIP7;1-2<br>NtNIP8;1-2<br>NtXIP1;1-2<br>NtXIP2;1-2	[FIV][TC]A[YF][LFW] [5]	NtNIP5;1<br>NtXIP1;1-2<br>NtXIP2;1-2
**CO_2**	FHTR [34]	NtPIP1;1-13<br>NtPIP2;1-21	SGGHINPAVT [38]	NtPIP1;1-13<br>NtPIP2;1-21<br>NtTIP3;1-4	GTGINPARSLG [13]	NtPIP1;1-8<br>NtPIP1;10<br>NtPIP1;12-13<br>NtPIP2;9-10	[MQ]SAFW [31]	NtPIP1;1-10<br>NtPIP1;12-13<br>NtPIP2;1-21
**H_2O_2**	[HFWI][IHV][ATG][VR] [71]	NtPIP1;1-13<br>NtPIP2;1-21<br>NtTIP1;1-9<br>NtTIP2;1-10<br>NtTIP3;1-4<br>NtTIP4;1-2<br>NtNIP1;1-2<br>NtNIP3;1-2<br>NtNIP4;1-6<br>NtXIP1;1-2	SG[GA]H[VLIF]NP[AV][VI][TS] [81]	NtPIP1;1-13<br>NtPIP2;1-21<br>NtTIP1;1-9<br>NtTIP2;1-10<br>NtTIP3;1-4<br>NtTIP4;1-2<br>NtTIP5;1-2<br>NtNIP1;1-2<br>NtNIP3;1-2<br>NtNIP4;1-5	G[AGT][SG][MI]NP[AG][VR][ASC][FL]G [68]	NtPIP1;1-8<br>NtPIP1;10<br>NtPIP1;12-13<br>NtPIP2;1-5<br>NtPIP2;7-21<br>NtTIP1;1-9<br>NtTIP2;1-10<br>NtTIP3;1-4<br>NtTIP4;1-2<br>NtNIP1;1-2	[TQFV][ASC]A[YF][WI] [41]	NtPIP1;12-13<br>NtPIP2;9-21<br>NtTIP1;1-9<br>NtTIP2;1-10<br>NtNIP3;2<br>NtNIP4;3-4<br>NtXIP1;1-2<br>NtXIP2;1-2

				NtNIP6;1-2<br>NtNIP7;1-2<br>NtNIP8;1-2<br>NtXIP1;1-2<br>NtXIP2;1-2		NtNIP3;1-2<br>NtNIP7;1-2<br>NtNIP8;1-2<br>NtXIP1;1-2<br>NtXIP2;1-2		
**NH_3**	[HW][IV][AG]R [26]	NtTIP2;1-10<br>NtNIP3;1-4<br>NtTIP4;1-2<br>NtNIP1;1-2<br>NtNIP3;1-2<br>NtNIP4;1-6	SGGH[VLF]NPAVT [24]	NtTIP1;1-9<br>NtTIP2;1-10<br>NtTIP4;1-2<br>NtTIP5;1-2<br>NtNIP4;1	G[GA]SMNPARS[FL]G [20]	NtTIP2;1-10<br>NtTIP4;1-2<br>NtNIP1;1-2<br>NtNIP3;1-2<br>NtNIP7;1-2<br>NtNIP8;1-2	[FT]SAY[LW] [21]	NtTIP1;1-9<br>NtTIP2;1-10<br>NtTIP4;1-2
**Si**	GSGR [1]	NtNIP2;1	SGAHMNPAVT [1]	NtNIP2;1	GGSMNPARTL[GA] [0]	0	[IL]TAYF [0]	0
**Urea**	[HGANI][ISV][AG][RVC] [30]	NtTIP1;1-9<br>NtTIP2;1-10<br>NtTIP3;1-4<br>NtTIP4;1-2<br>NtNIP5;1<br>NtNIP7;1-2<br>NtXIP1;1-2	SG[GA]H[ILVM]NP[AV][VI][TS] [70]	NtPIP1;1-13<br>NtPIP2;1-21<br>NtTIP1;1-9<br>NtTIP2;1-10<br>NtTIP3;1-4<br>NtTIP4;1-2<br>NtTIP5;1-2<br>NtNIP2;1<br>NtNIP6;1-2<br>NtNIP7;1-2<br>NtXIP1;1-2<br>NtXIP2;1-2	[GS][AG][SG]MNP[AV][RVC][TSC][LF]G [38]	NtTIP1;1-9<br>NtTIP2;1-10<br>NtTIP4;1-2<br>NtNIP1;1-2<br>NtNIP3;1-2<br>NtNIP5;1-3<br>NtNIP6;1-2<br>NtNIP7;1-2<br>NtNIP8;1-2<br>NtXIP1;1-2<br>NtXIP2;1-2	[MTLFVI][SATC]A[YF][WFL] [31]	NtTIP1;1-9<br>NtTIP2;1-10<br>NtTIP4;1-2<br>NtTIP3;1-4<br>NtNIP6;1-2<br>NtXIP1;1-2<br>NtXIP2;1-2
**As**	[GAW][VSAI][GA][RV] [15]	NtNIP1;1-2<br>NtNIP2;1<br>NtNIP3;1-2<br>NtNIP4;2-6<br>NtNIP5;1<br>NtNIP7;1-2<br>NtNIP8;1-2	SG[AC]H[LIVMF]NP[AS][VI]T [16]	NtNIP1;1-2<br>NtNIP2;1<br>NtNIP3;1-2<br>NtNIP4;2-6<br>NtNIP6;1-2<br>NtNIP7;1-2<br>NtNIP8;1-2	[GS][GA]SMNP[AV]R[ST][LI][AG] [20]	NtNIP1;1-2<br>NtNIP2;1<br>NtNIP3;1-2<br>NtNIP4;1-6<br>NtNIP5;1-3<br>NtNIP6;1-2<br>NtNIP7;1-2<br>NtNIP8;1-2	[LIFY][TS]AY[FILM] [16]	NtNIP1;1-2<br>NtNIP2;1<br>NtNIP3;1-2<br>NtNIP4;1-6<br>NtNIP5;1-3<br>NtNIP6;1-2

**Sb**	[AGT][IVSA][GA]R<br>[3]	NtNIP2;1<br>NtNIP5;1<br>NtNIP6;2	SG[AC]H[LM]NP[SA][VIT][TS]<br>[3]	NtNIP2;1<br>NtNIP6;1-2	[GS]-[GA]-SMNP[VA]R[TS]L[GA]<br>[13]	NtNIP1;1-2<br>NtNIP3;1-2<br>NtNIP5;1-3<br>NtNIP6;1-2<br>NtNIP7;1-2<br>NtNIP8;1-2	[FYIL][TS]AY[LMF]<br>[2]	NtNIP1;1-2
